# Author Correction: A method for Boolean analysis of protein interactions at a molecular level

**DOI:** 10.1038/s41467-023-41325-3

**Published:** 2023-09-06

**Authors:** Doroteya Raykova, Despoina Kermpatsou, Tony Malmqvist, Philip J. Harrison, Marie Rubin Sander, Christiane Stiller, Johan Heldin, Mattias Leino, Sara Ricardo, Anna Klemm, Leonor David, Ola Spjuth, Kalyani Vemuri, Anna Dimberg, Anders Sundqvist, Maria Norlin, Axel Klaesson, Caroline Kampf, Ola Söderberg

**Affiliations:** 1 Department of Pharmaceutical Biosciences, Science for Life Laboratory, Uppsala University, Biomedical Center, SE-751 24 Uppsala, Sweden; 2 Atlas Antibodies AB, Bromma, Sweden; 3 Faculty of Medicine, University of Porto, Porto, Portugal; 4 Differentiation and Cancer Group, Institute for Research and Innovation in Health (i3S) of the University of Porto/Institute of Molecular Pathology and Immunology of the University of Porto (Ipatimup), Porto, Portugal; 5 Department of Sciences, University Institute of Health Sciences (IUCS), CESPU, Gandra, Portugal; 6 Vi2, Department of Information Technology and SciLifeLab BioImage Informatics Facility, Uppsala University, Uppsala, Sweden; 7 Department of Immunology, Genetics and Pathology, Uppsala University, Rudbeck Laboratory, Uppsala, Sweden

**Keywords:** Proteomic analysis, Fluorescence imaging, Protein-protein interaction networks, Protein-protein interaction networks

Correction to: *Nature Communications* 10.1038/s41467-022-32395-w, published online 13 August 2022

The original version of this Article contained an error in Table 1. The correct version of the 3rd row (Circle part 2) of the 3rd column (Sequence (5’ -> 3’)) states ‘CGAGGTGCTTTTAGCACCTCGAAGTAAAGCTATCCACTGTCACCAACTACTAGATAAACGTCACACTTTTCGTGTGACG’

instead of the original, incorrect ‘CGAGGTGCTTTTAGCACCTCGAAGTAAAGCTATCCACTGTCACCAACTACTAGATAAACGTCACACTTTTCGTGTGAC’ where the final ‘G’ was omitted.

This has been corrected in both the PDF and HTML versions of the Article.

