# Supplementary material for: A method for Boolean analysis of protein interactions at a molecular level
Source: Nat Commun. 2022 Aug 13;13:4755. doi: 10.1038/s41467-022-32395-w (PMC9375095; doi:10.1038/s41467-022-32395-w)
Supplement: Supplementary file 2 — Reporting Summary [file 41467_2022_32395_MOESM2_ESM.pdf]

nature portfolio

Corresponding author(s): Ola Söderberg & Doroteya Raykova

Last updated by author(s): Jul 18, 2022

Reporting Summary

Nature Portfolio wishes to improve the reproducibility of the work that we publish. This form provides structure for consistency and transparency in reporting. For further information on Nature Portfolio policies, see our Editorial Policies and the Editorial Policy Checklist.

Statistics

For all statistical analyses, confirm that the following items are present in the figure legend, table legend, main text, or Methods section.

n/a	Confirmed	
☐	☒	The exact sample size (*n*) for each experimental group/condition, given as a discrete number and unit of measurement
☐	☒	A statement on whether measurements were taken from distinct samples or whether the same sample was measured repeatedly
☐	☒	The statistical test(s) used AND whether they are one- or two-sided<br>*Only common tests should be described solely by name; describe more complex techniques in the Methods section.*
☒	☐	A description of all covariates tested
☐	☒	A description of any assumptions or corrections, such as tests of normality and adjustment for multiple comparisons
☐	☒	A full description of the statistical parameters including central tendency (e.g. means) or other basic estimates (e.g. regression coefficient) AND variation (e.g. standard deviation) or associated estimates of uncertainty (e.g. confidence intervals)
☐	☒	For null hypothesis testing, the test statistic (e.g. *F*, *t*, *r*) with confidence intervals, effect sizes, degrees of freedom and *P* value noted<br>*Give P values as exact values whenever suitable.*
☒	☐	For Bayesian analysis, information on the choice of priors and Markov chain Monte Carlo settings
☒	☐	For hierarchical and complex designs, identification of the appropriate level for tests and full reporting of outcomes
☒	☐	Estimates of effect sizes (e.g. Cohen's *d*, Pearson's *r*), indicating how they were calculated

Our web collection on statistics for biologists contains articles on many of the points above.

Software and code

Policy information about availability of computer code

Data collection	No coding software was used. Software for image acquisition: Zen Blue 2 (widefield), Leica Application Suite X 3.7.4.23463 (confocal), Image Studio Lite v5.2.5 (gels)
Data analysis	Huygens Essential and CellProfiler v.4.1.3 pipeline is described in the Method section and code is provided in Supplementary Notes. Downstream analysis of MolBoolean and in situ PLA data used for binning, plotting and statistics is accessible at a provided link in Github (doi:10.5281/zenodo.6844631, see Software policy form and manuscript), along with an explanatory readme file.

For manuscripts utilizing custom algorithms or software that are central to the research but not yet described in published literature, software must be made available to editors and reviewers. We strongly encourage code deposition in a community repository (e.g. GitHub). See the Nature Portfolio guidelines for submitting code & software for further information.

Data

Policy information about availability of data

All manuscripts must include a data availability statement. This statement should provide the following information, where applicable:

- Accession codes, unique identifiers, or web links for publicly available datasets
- A description of any restrictions on data availability
- For clinical datasets or third party data, please ensure that the statement adheres to our policy

All data generated or analyzed during this study are included in this published article (and its supplementary files that include Supplementary Information and Source Data).

Field-specific reporting

Please select the one below that is the best fit for your research. If you are not sure, read the appropriate sections before making your selection.

☒ Life sciences ☐ Behavioural & social sciences ☐ Ecological, evolutionary & environmental sciences

For a reference copy of the document with all sections, see nature.com/documents/nr-reporting-summary-flat.pdf

Life sciences study design

All studies must disclose on these points even when the disclosure is negative.

Sample size	Based on what we have done before we performed each experiment in triplicate, and at least three images from each individual repeat were acquired, resulting in a total of minimum 9 images being analyzed per condition or assay. As we perform statistical analysis at single cell level, we obtain at least 100 cells per experimental condition, the size was estimated to be sufficient, Data was analyzed on per-cell basis, for all cell-based assays and the number of analyzed cells was not predetermined, but depended on how many cells were in frame in the analyzed microscopy images. Hence it varies for every experiment. Sample sizes (in number of cells analyzed) are provided in the figure legends wherever statistical comparisons were used. For tissues, analysis was performed per image frame.
Data exclusions	No data was excluded from the analyses.
Replication	Each experiment was replicated three times.
Randomization	For each experiment, images were acquired from three random regions of the well or tissue slide. Cell densities were the same for treated and non-treated cell cultures.
Blinding	The same person performed all steps in the experiment. We selected random fields of view where images were taken, based on Hoechst 33342 staining which can be considered as blinded (as we then didn´t know the MolBoolean staining)

Reporting for specific materials, systems and methods

We require information from authors about some types of materials, experimental systems and methods used in many studies. Here, indicate whether each material, system or method listed is relevant to your study. If you are not sure if a list item applies to your research, read the appropriate section before selecting a response.

Materials & experimental systems

n/a	Involved in the study	
☐	☒	Antibodies
☐	☒	Eukaryotic cell lines
☒	☐	Palaeontology and archaeology
☒	☐	Animals and other organisms
☐	☒	Human research participants
☒	☐	Clinical data
☒	☐	Dual use research of concern

Methods

n/a	Involved in the study	
☒	☐	ChIP-seq
☒	☐	Flow cytometry
☒	☐	MRI-based neuroimaging

Antibodies

Antibodies used	All antibodies used, along with relevant information on their supplier, product numbers and concentration/dilution they were used at is detailed in Table 2. For the antibody conjugates the concentration used is specified in M&M.
Validation	All antibodies, except these provided by Atlas Antibodies, were validated in-house by Western blotting and immunofluorescence.<br><br>These antibodies have also been validated by Western blotting by the respective manufacturer. Anti-E-cadherin (BD Transduction Laboratories, #610182) was additionally indicated for use in immunoprecipitation, immunohistochemistry, immunofluorescence, chIP, and chIP-seq by the manufacturer. Anti-mesothelin (Thermo Fisher Scientific, MA5-16378) and anti-Lamin A/C (Cell signaling #2032) were additionally validated with immunohistochemistry in paraffin-embedded tissues by the supplier. Anti-calreticulin (Cell signaling #12238) was additionally validated with flow cytometry by the supplier. Anti-PDGFR-b (Cell signaling #3169) was additionally validated with immunoprecipitation and immunohistochemistry in paraffin-embedded tissue sections by the supplier. Anti-clathrin (Abcam, ab2731) and anti-GM130 (BD Biosciences, #610822) were additionally validated with immunohistochemistry by the supplier.<br><br>Regarding all primary antibodies provided by Atlas Antibodies:<br>All Triple A Polyclonals and PrecisA Monoclonals offered by Atlas Antibodies are validated and characterized in each application that each specific antibody is approved for (IHC, ICC-IF, WB). For IHC, more than 40 normal tissues and 20 cancer tissues were used for validation and each Triple A polyclonal antibody is accompanied with a large number of images on the Human Protein Atlas showing the result of these validation assays, each manually annotated by researchers. For ICC and WB, a selection of suitable cell lines

ensuring endogenous expression of the target gene (based on HPA Protein Atlas RNA expression data in cell lines) were used in the antibody characterization/validation. The validation methods stated above are called “standard validation”. In addition to the extensive validation and characterization always performed for all our antibodies, we also perform application-specific “Enhanced Validation”. The enhanced validation follows the guidelines proposed by the International Working Group for Antibody Validation (IWGAV) in Nature Methods. At least one of the 5 conceptual pillars proposed in this paper must be used for an antibody to be given the “enhanced validation” stamp.

Eukaryotic cell lines

Policy information about cell lines

Cell line source(s)	MCF7 (ECCAC, cat. no. 86012803) and U2OS (ECCAC, cat. no. 92022711) were obtained from the ECCAC General Cell Collection, HaCaT (DSMZ, ACC-771) and BJ hTERT (ATCC, CRL-4001) were a kind gift from Prof. Aristidis Moustakas from Uppsala University. AGS clones were a kind gift from Assoc. Prof. Raquel Seruca, University of Porto.
Authentication	None of the cell lines were authenticated in-house
Mycoplasma contamination	All cell lines were negative for mycoplasma
Commonly misidentified lines (See ICLAC register)	No commonly misidentified lines according to the ICLAC register were used

Human research participants

Policy information about studies involving human research participants

Population characteristics	The ovarian cancer case used in the study is a paradigm of a population of serous ovarian carcinomas with clinical indication for primary oophorectomy.
Recruitment	The single human tissue specimen used in the study was selected from a series of consecutive high grade serous ovarian carcinomas, operated at Centro Hospital de São João.
Ethics oversight	Ethical Committee from Centro Hospital de São João

Note that full information on the approval of the study protocol must also be provided in the manuscript.
