# Supplementary material for: A method for Boolean analysis of protein interactions at a molecular level
Source: Nat Commun. 2022 Aug 13;13:4755. doi: 10.1038/s41467-022-32395-w (PMC9375095; doi:10.1038/s41467-022-32395-w)
Supplement: Supplementary file 4 — Source Data [file 41467_2022_32395_MOESM4_ESM.zip › Original uncropped blot images.pdf]

Original Images for Blots, PDIA3 knock-down (Fig. 7, Suppl. Fig. 5)

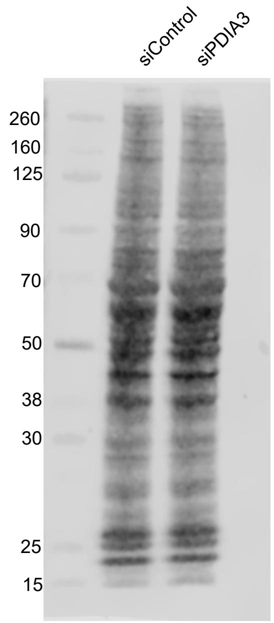

Fig. 7, blot 1, channel 1
Total protein

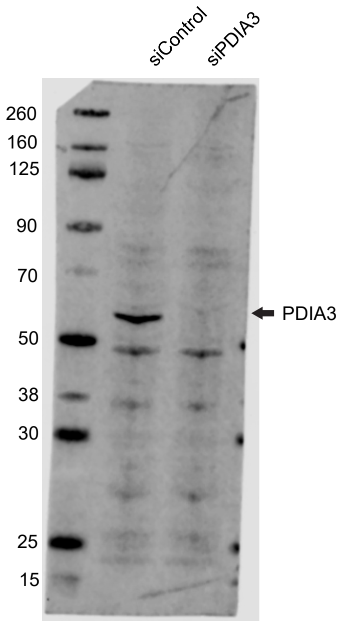

Fig. 7, blot 1,channel 2
