# Supplementary material for: A method for Boolean analysis of protein interactions at a molecular level
Source: Nat Commun. 2022 Aug 13;13:4755. doi: 10.1038/s41467-022-32395-w (PMC9375095; doi:10.1038/s41467-022-32395-w)
Supplement: Supplementary file 1 — Supplementary Information [file 41467_2022_32395_MOESM1_ESM.pdf]

MolBoolean: A Boolean Analysis of Protein-Protein Interactions at a Molecular Level

Supplementary Notes

1. Oligonucleotide Design

At the foundation of the MolBoolean design is the information receiver circle: it is essential for the barcoding of the signal and for signal amplification via RCA. Because of the many functional regions that it needs to contain, its single DNA strand has to be rather long, which makes it difficult to synthesize as a single molecule. Therefore, the circle is initially designed as two DNA oligonucleotides of different lengths, which are later ligated together (Table 1). Circle part 1 is 114 nucleotides (nt) long and has a sequence that forms incomplete hairpin structures both on its 5' and 3' ends. Circle part 2 is 79 nt long and has 5' and 3' regions that can bind in a complementary fashion to circle part 1 so as to complete the hairpins. Thus, a disconnected circle with two complete hairpin structures, to which each circle part contributes, is formed. These double-stranded hairpin regions serve as a template for T4 ligase which joins the two circle parts into a fully closed circularized molecule.

The information receiver circle has two important characteristics: its sequence, and its length. The sequence contains recognition regions A and B where the arms of the MolBoolean probes can hybridize specifically. Despite their differing sequences, each of these regions contains the same restriction site specific for the Nt.BsmAI nickase. The circle length has a direct correlation with the efficiency of RCA during signal amplification[1]. We therefore designed our circle-forming oligonucleotides to be as short as possible, but at a length that provides amplification maxima for all conditions (i.e. when either one or both tags get integrated in the circle).

The MolBoolean proximity probes consist of affinity purified donkey anti-mouse or anti-rabbit IgG antibodies, or of primary antibodies, covalently linked to appropriately modified arms, depending on the conjugation method used (Table 1). There are multiple alternative ways in which the arms can be linked to the antibodies (applicable to both primary and secondary proximity probes), for example based on hydrazine chemistry, click chemistry or photo-crosslinking (Light-Activated Site-Specific Conjugation), such as in the oYo-Link conjugation technology, etc. These methods achieve slightly different results. For example, in hydrazine chemistry (SANH) conjugation one antibody molecule can be labelled with multiple oligonucleotides, since SANH reacts with primary amines (ε-amino group of lysine residues) on the protein, creating HyNic-activated molecules that can on their turn react with the aldehyde modifications on oligonucleotides. In contrast, oYo-Link conjugation generates site-specific labelling in the Fc region of antibodies based on non-damaging black light photo-crosslinking, leading to a maximum of two labels per antibody. Arm A is conjugated to the anti-mouse IgG or primary antibody, and arm B to the anti-rabbit IgG or primary antibody, creating probe A and probe B respectively (Fig. 1a). Each arm consists of a hinge part that provides mobility, and a recognition part, which is complementary to one of the two recognition regions in the circle. Therefore, the recognition part of the arm also contains the Nt.BsmAI motif. Importantly, the arm contains a stem-and-loop structure positioned just before this motif, which is essential for the next step of tag incorporation. The loop and the majority of the stem sequence in arm A are complementary to tag A, the same goes for arm B and tag B.

The detection oligonucleotides were designed to have identical sequences as their corresponding tags, with the exception that they are blocked in the 3’ end (to prevent accidental RCA priming) and labeled with a fluorophore in their 5’ end (Table 1).

Wells	1	2	3	4	5	6	7	8	9	10	11	12	13	14
Circle	+	-	-	-	-	+	+	+	+	+	+	+	+	+
Arm A	-	+	-	-	-	-	+	-	+	+	+	+	-	+
Arm B	-	-	+	-	-	-	-	+	+	+	+	+	+	-
Tag A	-	-	-	+	-	-	-	-	-	+	-	+	+	-
Tag B	-	-	-	-	+	-	-	-	-	-	+	+	-	+
Nickase	-	-	-	-	-	+	+	+	+	+	+	+	+	+
Ligase	-	-	-	-	-	-	-	-	-	+	+	+	+	+

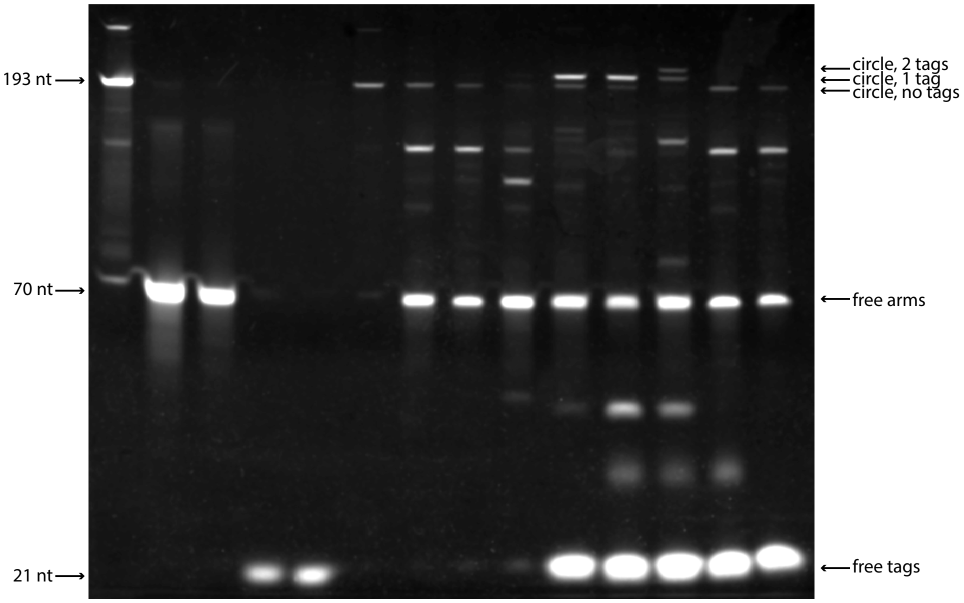

Suppl. Fig. 1. *In solution* testing of the MolBoolean specificity. TBE-Urea gel showing
migration of MolBoolean oligonucleotides after *in solution* reactions of Nt.BsamA.I digestion and ligation. The constituents of the reactions in each well are marked with a '+' under the corresponding well number. The individual components of the system were loaded on the gel as size references (wells 1-5). The single-stranded circle does not get digested in the absence of arm hybridization (well 6), but is nicked after either or both of the arms hybridize to it (wells 7-9). Following circle digestion, tags are successfully incorporated and ligated into the circle (wells 10-12, higher migrating bands), unless a mismatched combination of arm and tag is introduced, i.e. arm A with tag B or vice versa (wells 13, 14). Representative image is shown. Data collected from three independent experiments.

2. Additional No-Interaction Control Example

We utilized MolBoolean to detect MT-CO1 (also known as COX1) and GM130 in MCF7 cells (Suppl. Fig. 3c, d). MT-CO1 is a mitochondrial DNA-encoded protein and the main unit of the cytochrome c oxidase complex located in the inner mitochondrial membrane[2,3]. GM130, on the other hand, is a marker for the Golgi apparatus and is thought to have a role in maintaining its structure and in protein transport from the endoplasmic reticulum to the Golgi apparatus, among others[4,5] (https://www.proteinatlas.org/ENSG00000167110-GOLGA2). The two proteins are abundantly expressed in MCF7 cells (see proteinatlas.org), but are not expected to form a complex. IF co-stain was performed in parallel to MolBoolean and confirmed the disparate subcellular localizations of MT-CO1 and GM130 corresponding to what has been described in literature (Suppl. Fig. 3d). The *in situ* PLA stains were nearly blank when either one of the two primary antibodies was omitted (Suppl. Fig. 3c), and a small number of RCPs per cell was detected in the presence of both primary antibodies (Suppl. Fig. 3c). The MolBoolean quantification showed a higher number of detected complexes compared to *in situ* PLA. However, this was still a very small number compared the high levels of free GM130 and MT-CO1.

3. Direct conjugation

When performing MolBoolean it is possible to skip secondary antibodies altogether and use probes based on direct primary antibody conjugation instead: an approach which lowers the distance requirement for dual signal generation. The results obtained by directly conjugating E-cadherin and β-catenin to arms A and B respectively in TGFβ1-treated cells and controls were comparable to those obtained with the use of secondary probes (Suppl. Fig. 5b and Fig. 5a, respectively). For details on how direct conjugation was performed in this experiment, please see Methods (sub-section oYo-Link conjugation).

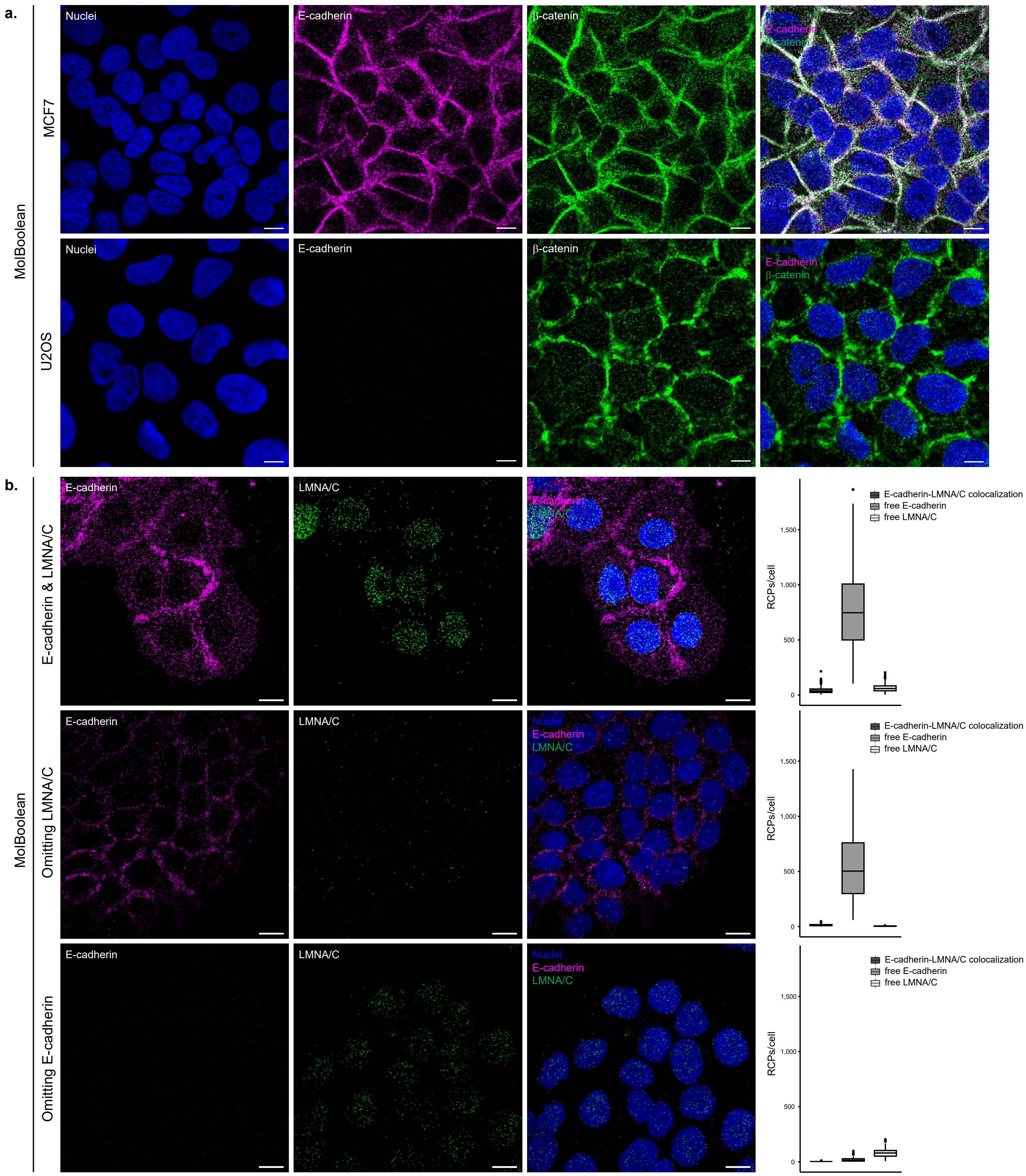
a.
MolBoolean
MCF7
U2OS
Nuclei
E-cadherin
β-catenin
Nuclei
E-cadherin
β-catenin
b.
MolBoolean
E-cadherin & LMNA/C
Omitting LMNA/C
Omitting E-cadherin
E-cadherin
LMNA/C
Nuclei
E-cadherin
LMNA/C
RCPs/cell
1,500
1,000
500
0
E-cadherin-LMNA/C colocalization
free E-cadherin
free LMNA/C

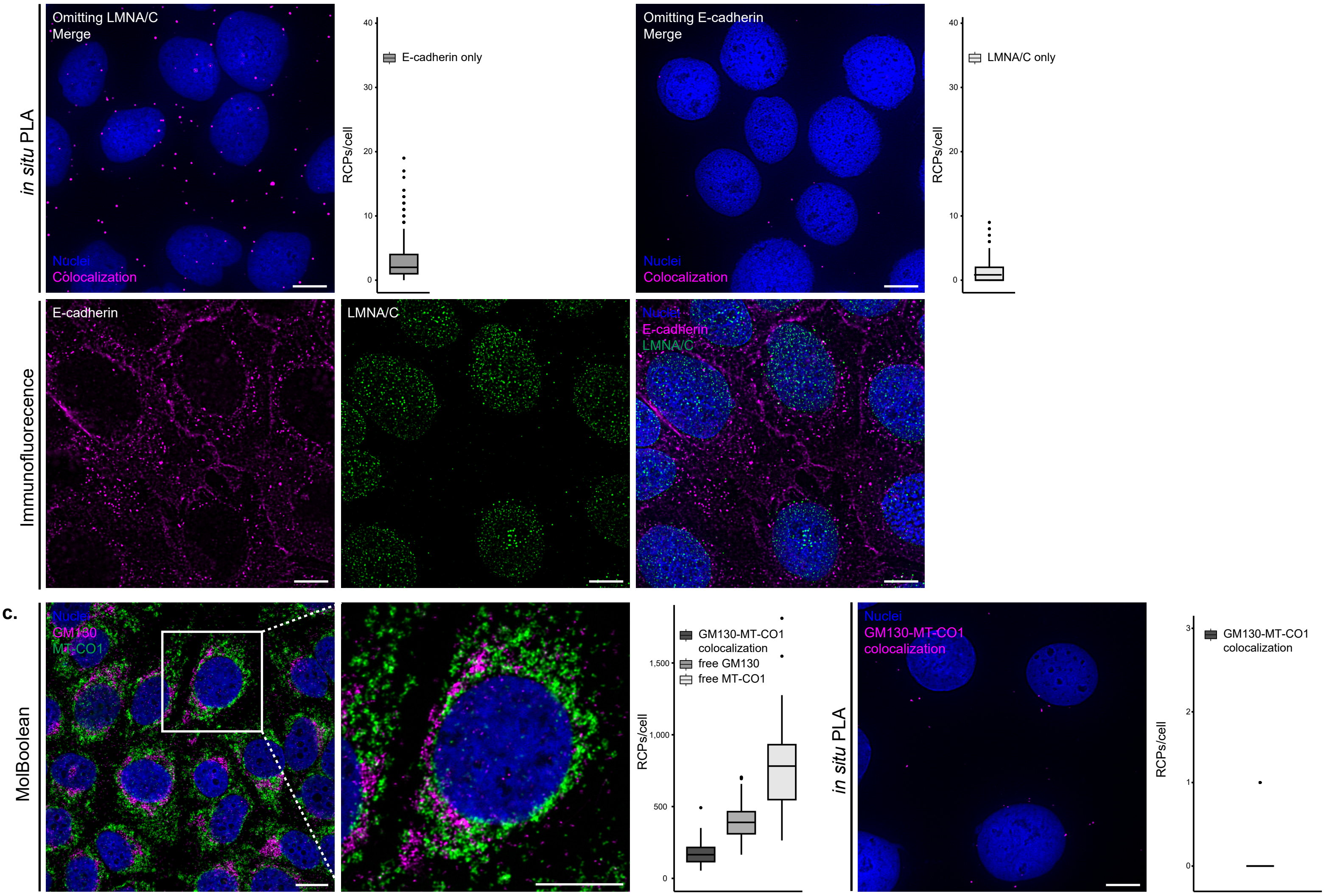
in situ PLA
Omitting LMNA/C
Merge
Nuclei
Colocalization
RCPs/cell
E-cadherin only
0
10
20
30
40
Omitting E-cadherin
Merge
Nuclei
Colocalization
LMNA/C only
Immunofluorescence
E-cadherin
LMNA/C
Nuclei
E-cadherin
LMNA/C
c.
MolBoolean
Nuclei
GM130
MT-CO1
GM130-MT-CO1
colocalization
free GM130
free MT-CO1
RCPs/cell
0
500
1,000
1,500
in situ PLA
Nuclei
GM130-MT-CO1
colocalization
GM130-MT-CO1
colocalization
RCPs/cell
0
1
2
3

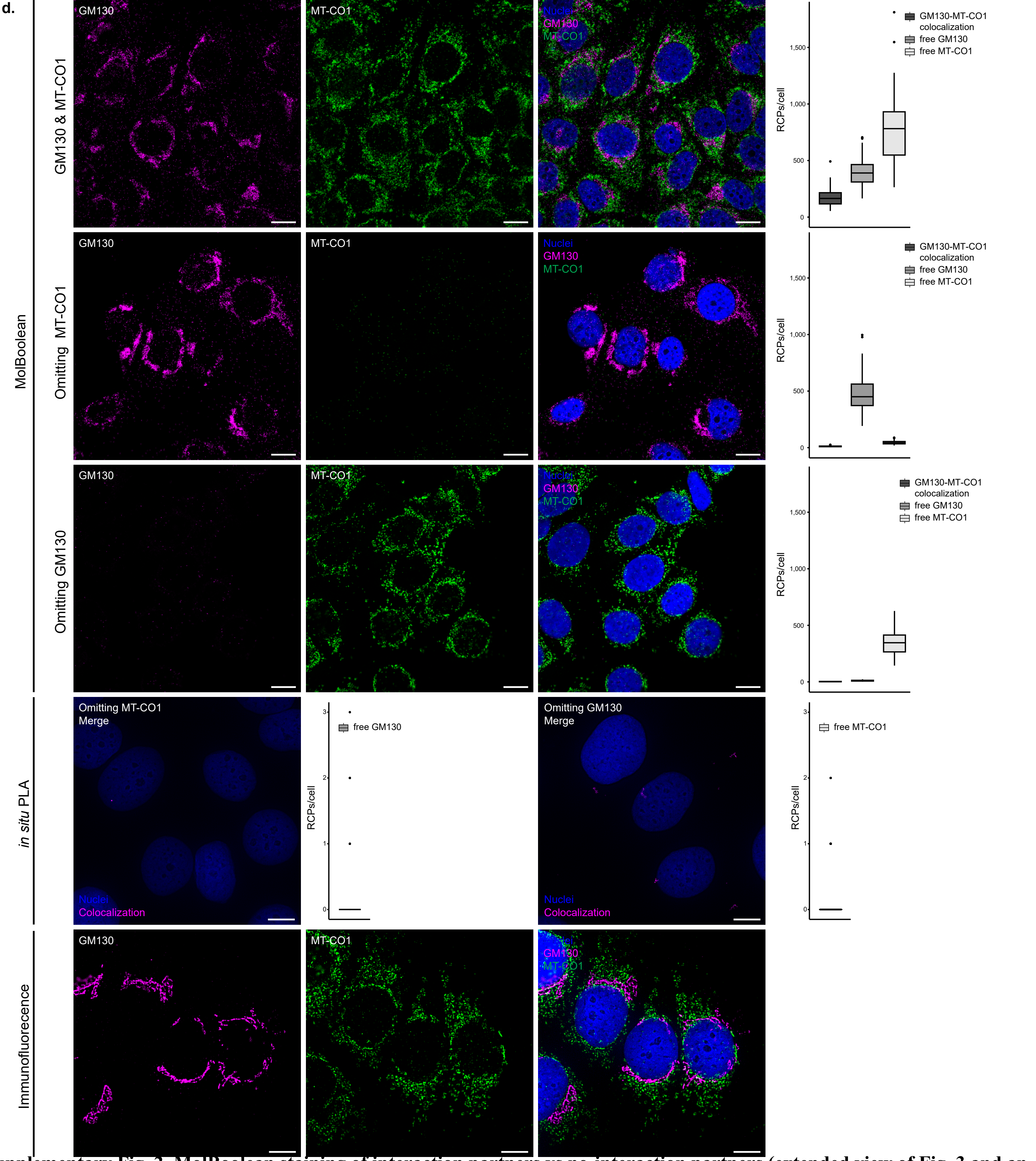

Supplementary Fig. 2. MolBoolean staining of interaction partners vs no-interaction partners (extended view of Fig. 3 and an additional no-interaction control). (a) Extended view of Fig. 3a: E-cadherin and β-catenin co-stain in MCF7 and U2OS cells. Both proteins are expressed in MCF7, but only β-catenin is expressed in U2OS. E-cadherin is shown in magenta, β-catenin in green, E-cadherin-β-catenin complex in white, nuclei in blue. **(b)** Extended view of Fig. 3b, omitting controls and IF validation of co-stains against no-interaction partners E-cadherin and Lamin A/C in HaCaT cells. MolBoolean and IF signals for E-cadherin shown in magenta, LMNA/C in green and nuclei in blue. *in situ* PLA signals for E-cadherin-LMNA/C colocalization are shown in magenta and nuclei n blue. **(c)** MolBoolean and i*n situ* PLA staining against non-interacting proteins GM130 and MT-CO1 in MCF7 cells. MolBoolean signals for GM130 are shown in magenta, MT-CO1 in green, and nuclei in blue. *in situ* PLA signals for GM130-MT-CO1 colocalization are shown in magenta. **(d)** Extended view of (c), omitting controls and IF validation of co-stains against GM130 and MT-CO1 in MCF7 cells. MolBoolean and IF signals for GM130 are shown in magenta, MT-CO1 in green, and nuclei in blue. *in situ* PLA signals for GM130-MT-CO1 colocalization are shown in magenta.
White frames depict an area shown in enlarged view in the following panel. Quantification of protein complexes and free proteins (MolBoolean) or protein complexes only (i*n situ* PLA) shown as number of RCPs per cell. Scale bars = 10 µm. Data collected from three independent experiments. Box plots show median, Q1 to Q3 range, lower and upper whiskers at maximum 1.5 times the inter-quartile range. Outliers shown as solid circles. Source data are provided as a Source Data file.

a.

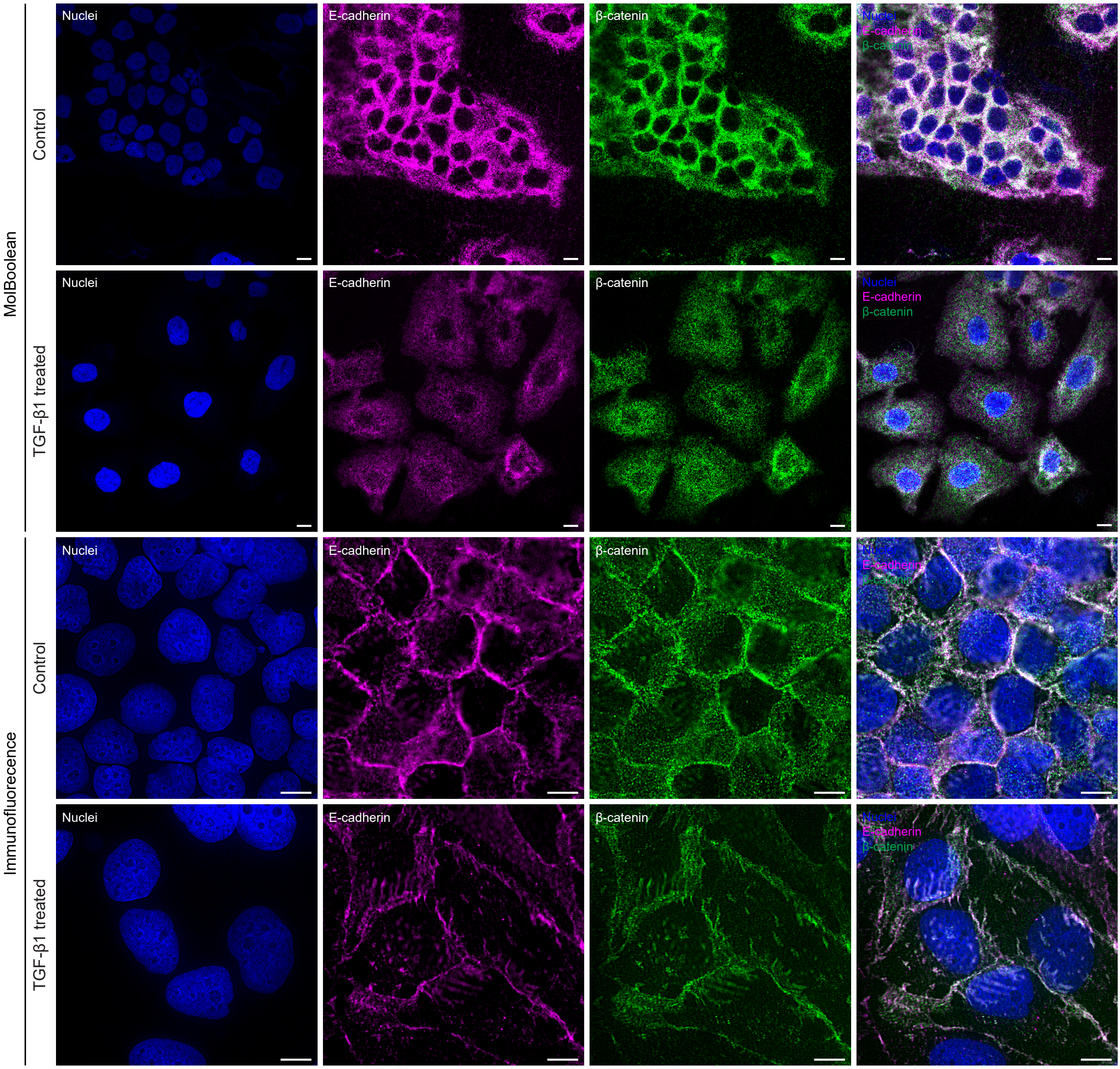

MolBoolean
Immunofluorecence
Control
TGF-β1 treated
Nuclei
E-cadherin
β-catenin

b.

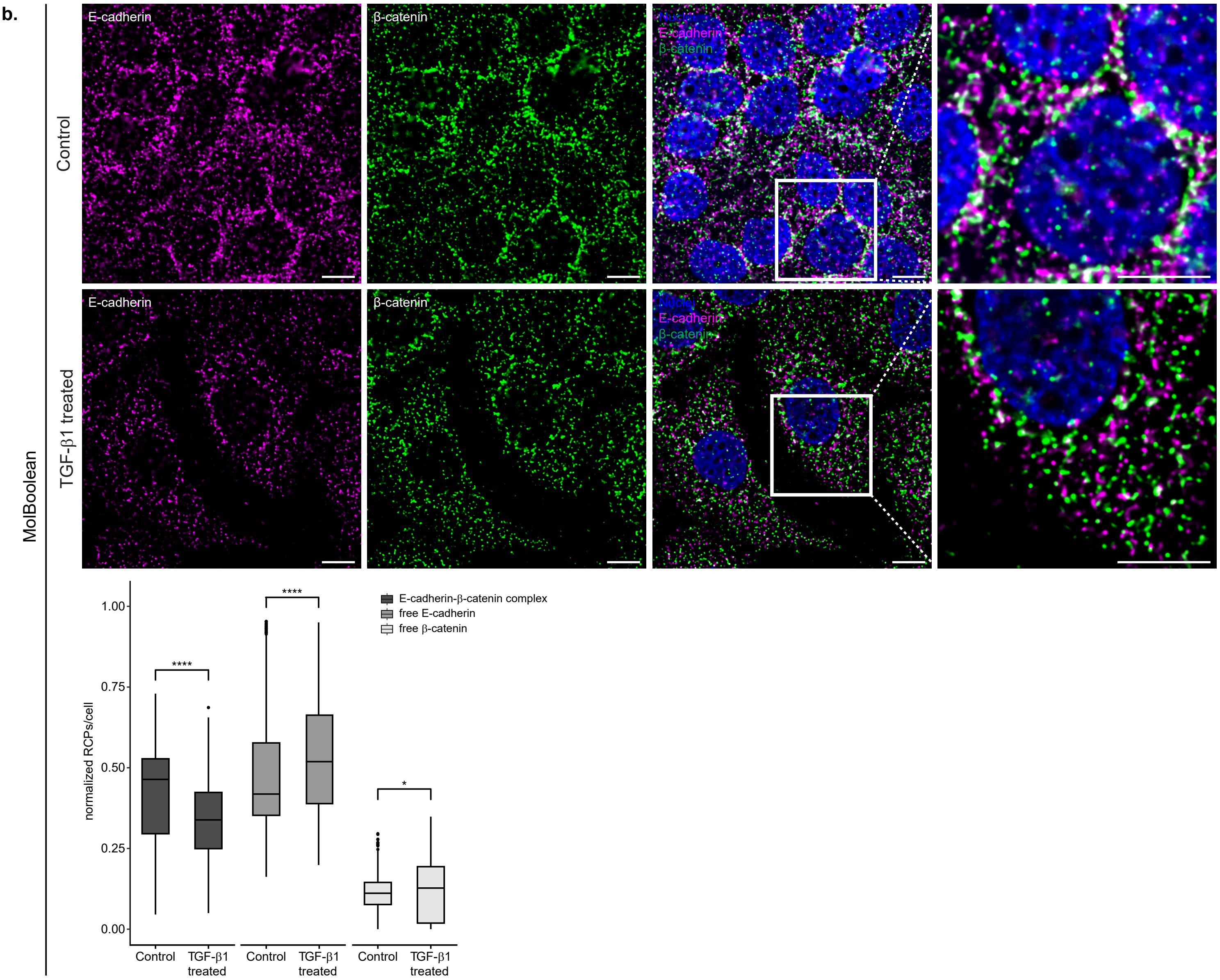

MolBoolean
Control
TGF-β1 treated
E-cadherin
β-catenin
Nuclei
E-cadherin
β-catenin
E-cadherin-β-catenin complex
free E-cadherin
free β-catenin
normalized RCPs/cell
1.00
0.75
0.50
0.25
0.00
****
****
*
Control
TGF-β1 treated
Control
TGF-β1 treated
Control
TGF-β1 treated

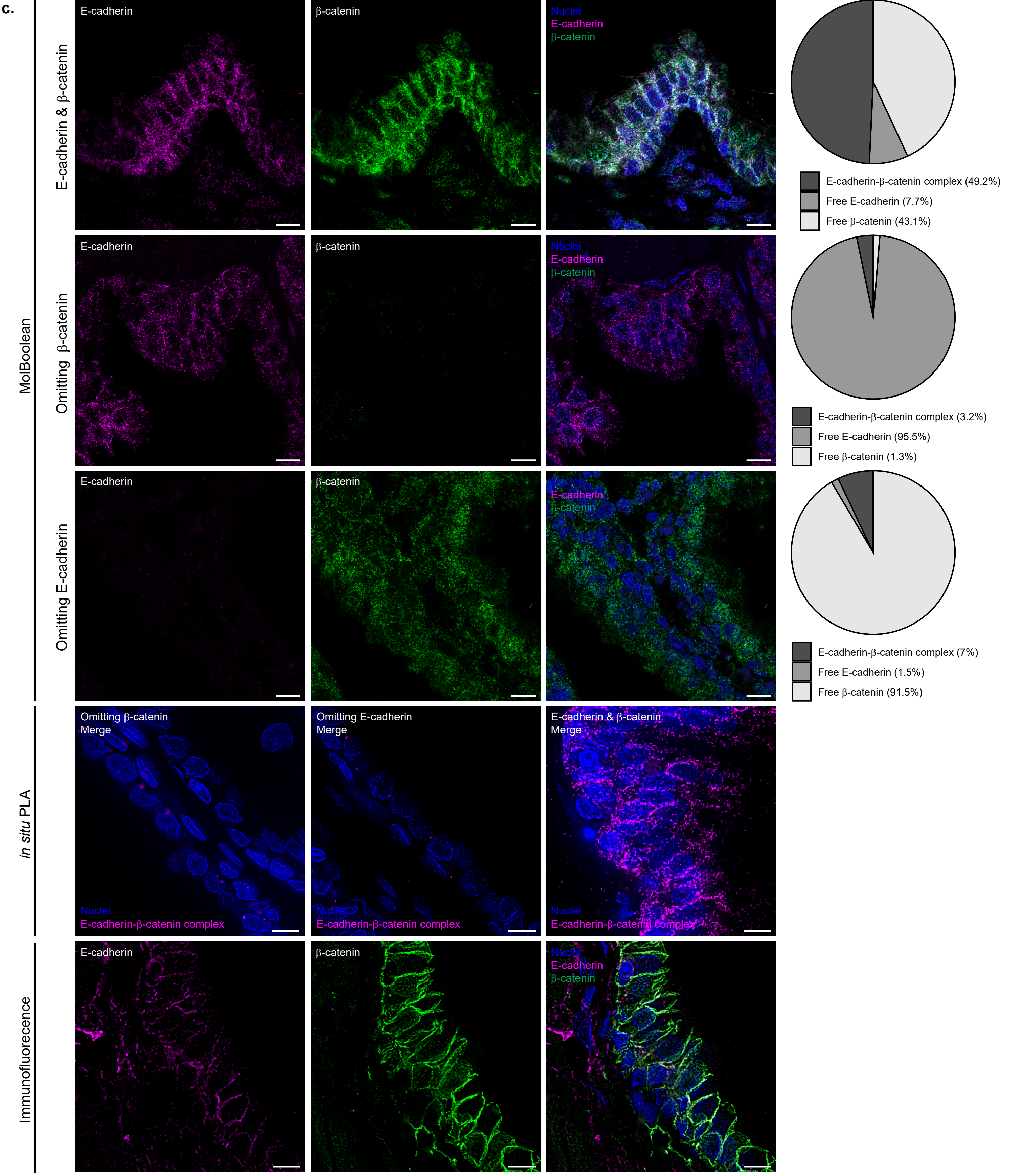

Supplementary Fig. 3. MolBoolean and *in situ* PLA staining of E-cadherin and β-catenin in ligand-inducible interactions in fixed cells, or in FFPE tissue sections (extended view of Fig. 5 and IF validation of ligand-inducible interactions; omitting controls and IF validation of FFPE tissue co-stains. (a-b) E-cadherin and β-catenin co-stains in HaCaT cells, in control conditions (top) or after TGF-β1 treatment (bottom), using secondary (a) or directly conjugated probes (b) ($p = 1.81e-23$; $p = 7.16e-12$; $p = 0.02$ for E-cadherin-β-catenin complex, free E-cadherin and free β-catenin respectively. **(c)** E-cadherin and β-catenin co-stains in FFPE prostate tissue sections.

MolBoolean and signals for E-cadherin is shown in magenta, β-catenin in green, E-cadherin-β-catenin complex in white, nuclei in blue. *in situ* PLA signals for E-cadherin-β-catenin complex shown in magenta and nuclei shown in blue.

White frames depict an area shown in enlarged view in the following panel. MolBoolean quantification shown as number of RCPs per cell (b) or in percentage of RCPs in each category (free protein A, free protein B and AB complex) per frame (c). Scale bars = 10μm. $n_{control} = 371$, $n_{treated} = 113$ cells (a); $n_{control} = 690$, $n_{treated} = 553$ cells (b); data collected from three independent experiments . Two-sided Wilcoxon rank sum test was used to analyze statistical variance. Box plots show median, Q1 to Q3 range, lower and upper whiskers at maximum 1.5 times the interquartile range. Outliers shown as solid circles. $p < 0.05$ (*), $p < 0.0001$ (****). Source data are provided as a Source Data file.

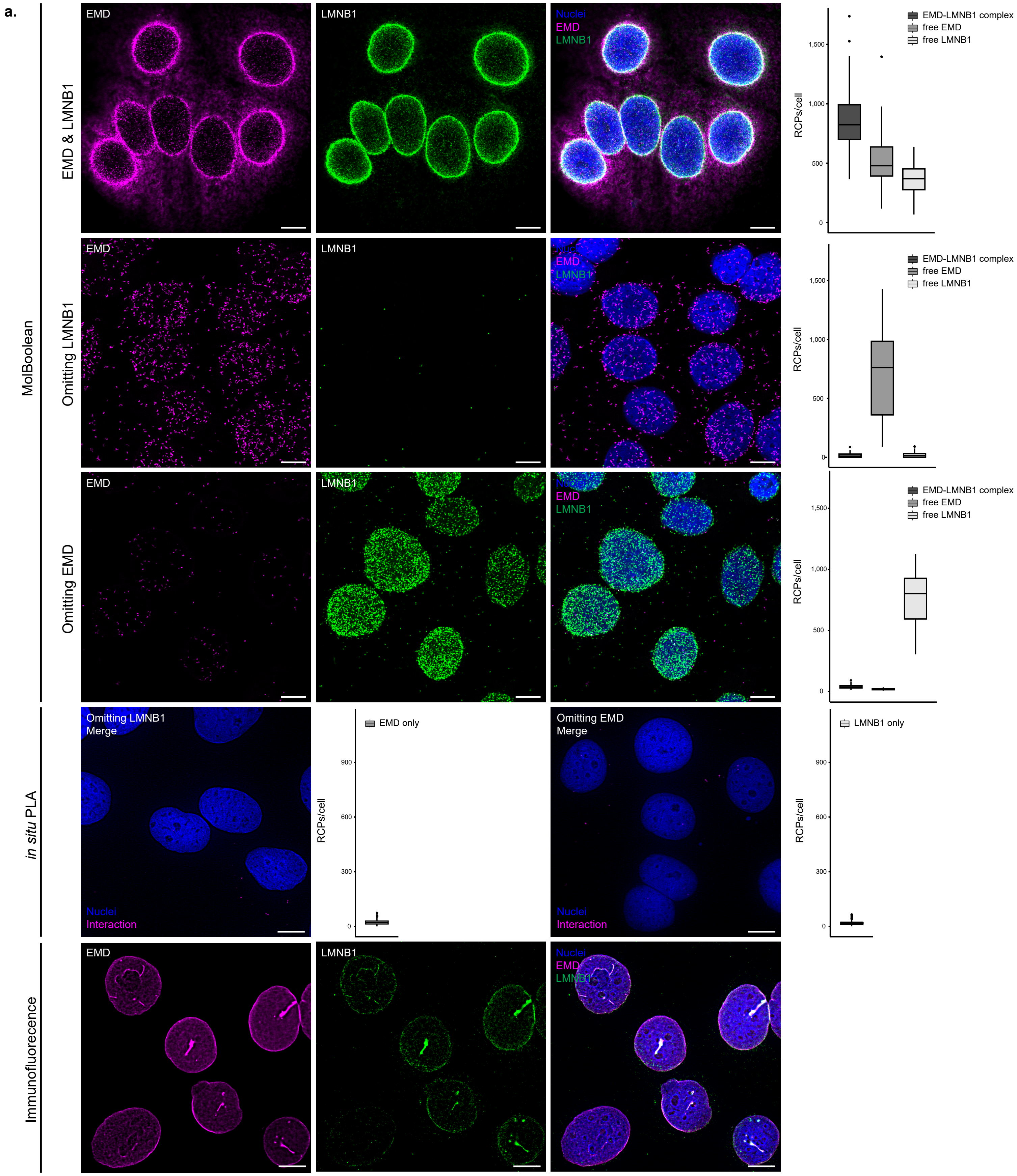

a.
MolBoolean
EMD & LMNB1
Omitting LMNB1
Omitting EMD
in situ PLA
Immunofluorecence
EMD
LMNB1
Nuclei
EMD
LMNB1
EMD-LMNB1 complex
free EMD
free LMNB1
RCPs/cell
1,500
1,000
500
0
Omitting LMNB1
Merge
Nuclei
Interaction
EMD only
900
600
300
0
Omitting EMD
Merge
LMNB1 only

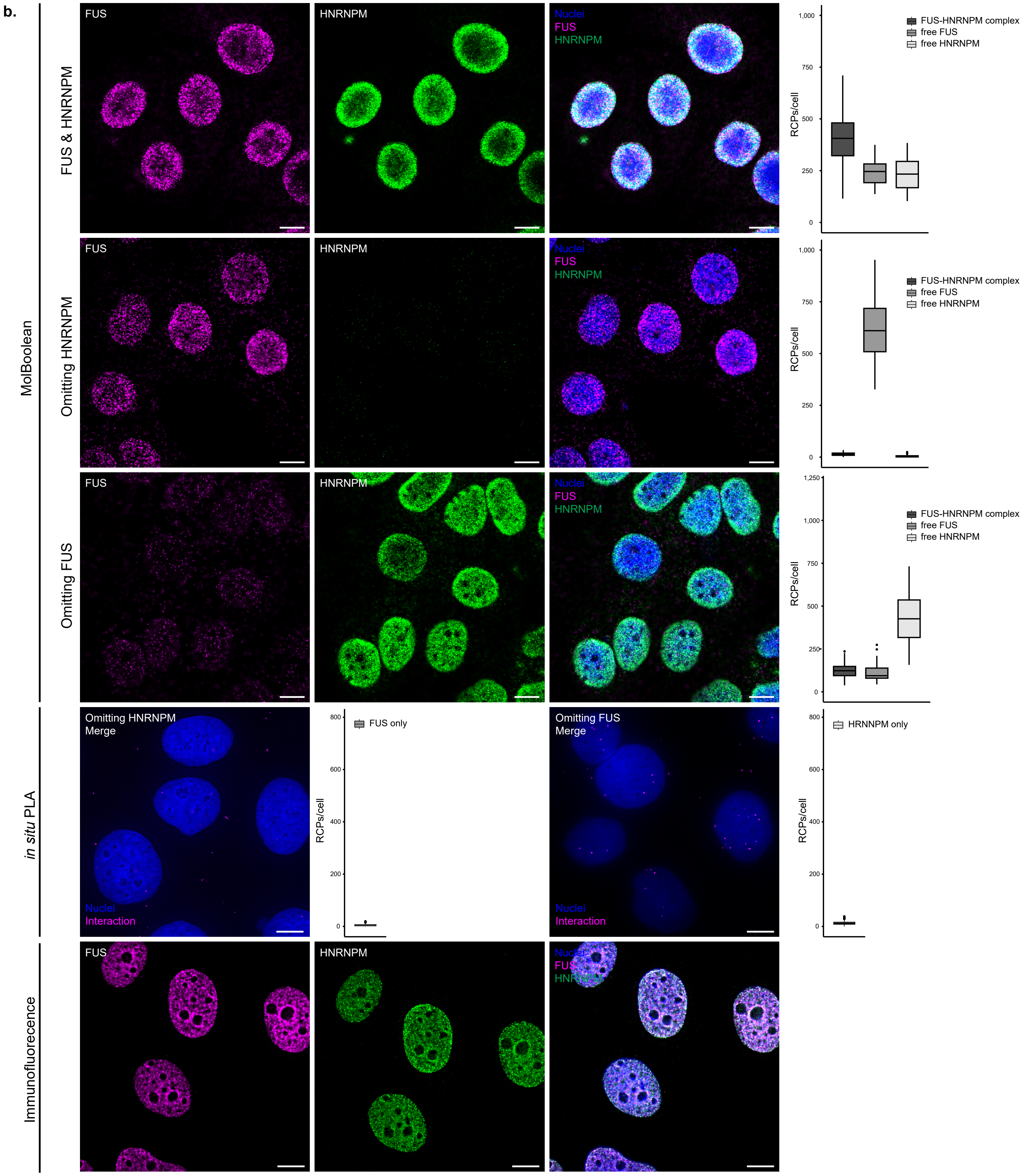

Supplementary Fig. 4. MolBoolean and *in situ* PLA staining of nuclear proteins in MCF7 cells (extended view of Fig. 6, omitting controls and, IF validation). (a) EMD and LMNB1 co-stain in MCF7 cells. MolBoolean and IF signals for EMD shown in magenta, LMNB1 in green, EMD-LMNB1 complex in white, and nuclei in blue. *in situ* PLA signals for EMD-LMNB1 complex shown in magenta, and nuclei in blue. **(b)** FUS and HNRNPM co-stain in MCF7 cells. MolBoolean and IF signals for FUS shown in magenta, HNRNPM in green, FUS-HNRNPM complex in white, and nuclei in blue. *in situ* PLA signals for FUS-HNRNPM complex shown in magenta, and nuclei in blue.

MolBoolean quantification shown as number of RCPs per cell. Scale bars = 10µm. Data collected from three independent experiments. Box plots show median, Q1 to Q3 range, lower and upper whiskers at maximum 1.5 times the interquartile range. Outliers shown as solid circles. Source data are provided as a Source Data file.

a.

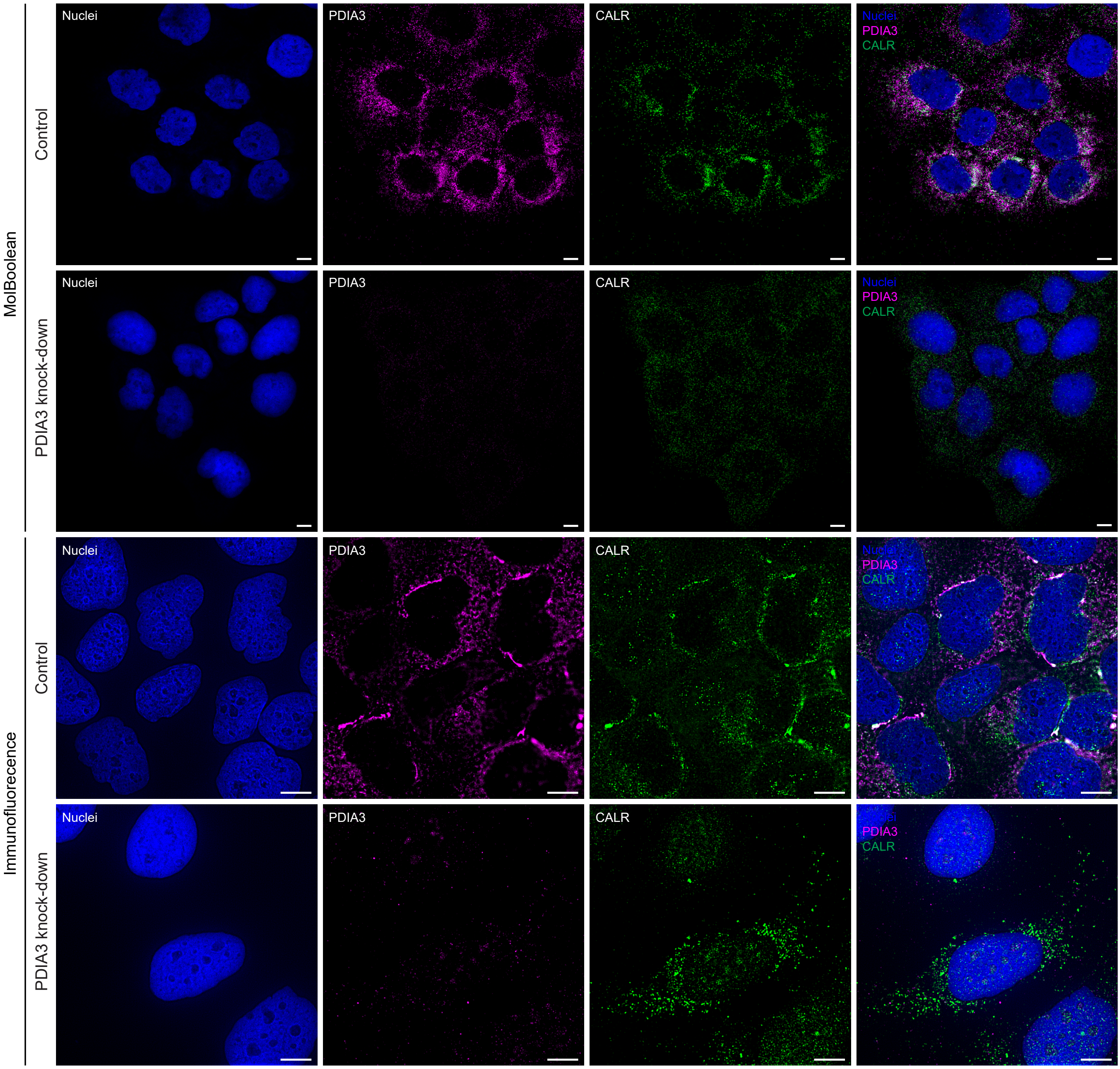

MolBoolean
Control
PDIA3 knock-down
Immunofluorecence
Nuclei
PDIA3
CALR

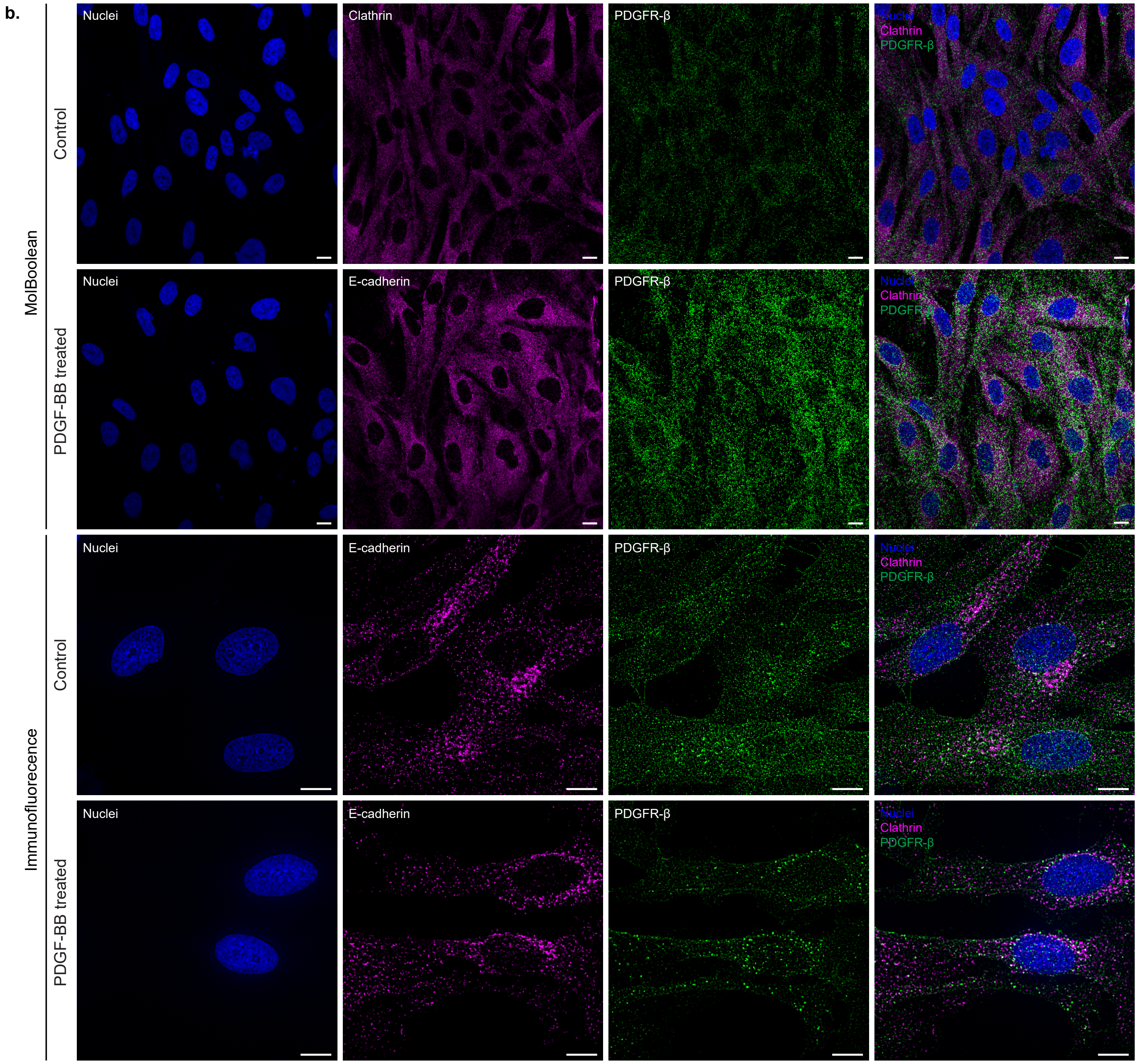

Supplementary Fig. 5. MolBoolean staining in dynamic conditions (extended view of Fig. 7 and, IF validation of co-stains with one knocked-down interaction partner and, ligand-inducible interactions). (a) PDIA3 and CALR co-stain in HaCaT cells in control (top) or siPDIA3-silenced cells (bottom). MolBoolean and IF signals for PDIA3 shown in magenta, CALR in green, PDIA3-CALR complex in white, and nuclei in blue. **(b)** Clathrin and PDGFR-β co-stain in BJ-hTERT cells, in control conditions (top) or after PDGF-BB treatment (bottom). MolBoolean and IF signals for Clathrin shown in magenta, PDGFR-β in green, Clathrin-PDGFR-β complex in white, and nuclei in blue.
Data collected from three independent experiments.

4. Additional FFPE Tissue Staining Examples

To provide even more staining examples in clinically relevant tissue samples, we performed two additional experiments. In Suppl. Fig. 8c, we applied MolBoolean to detect Mucin-16 (MUC16) and Mesothelin (MSLN) that are well-known to bind selectively as the first step of ovarian tumor invasion of the peritoneum[6,7] in ovarian cancer tissue. We successfully observed the characteristic pattern of interaction around the edges of tissue lumina as well as both free Mucin-16 and Mesothelin. Suppl. Fig. 8d demonstrates the FUS-HNRNPM interaction that we showed in cells in Fig. 6b, but this time in prostate tissue.

a.

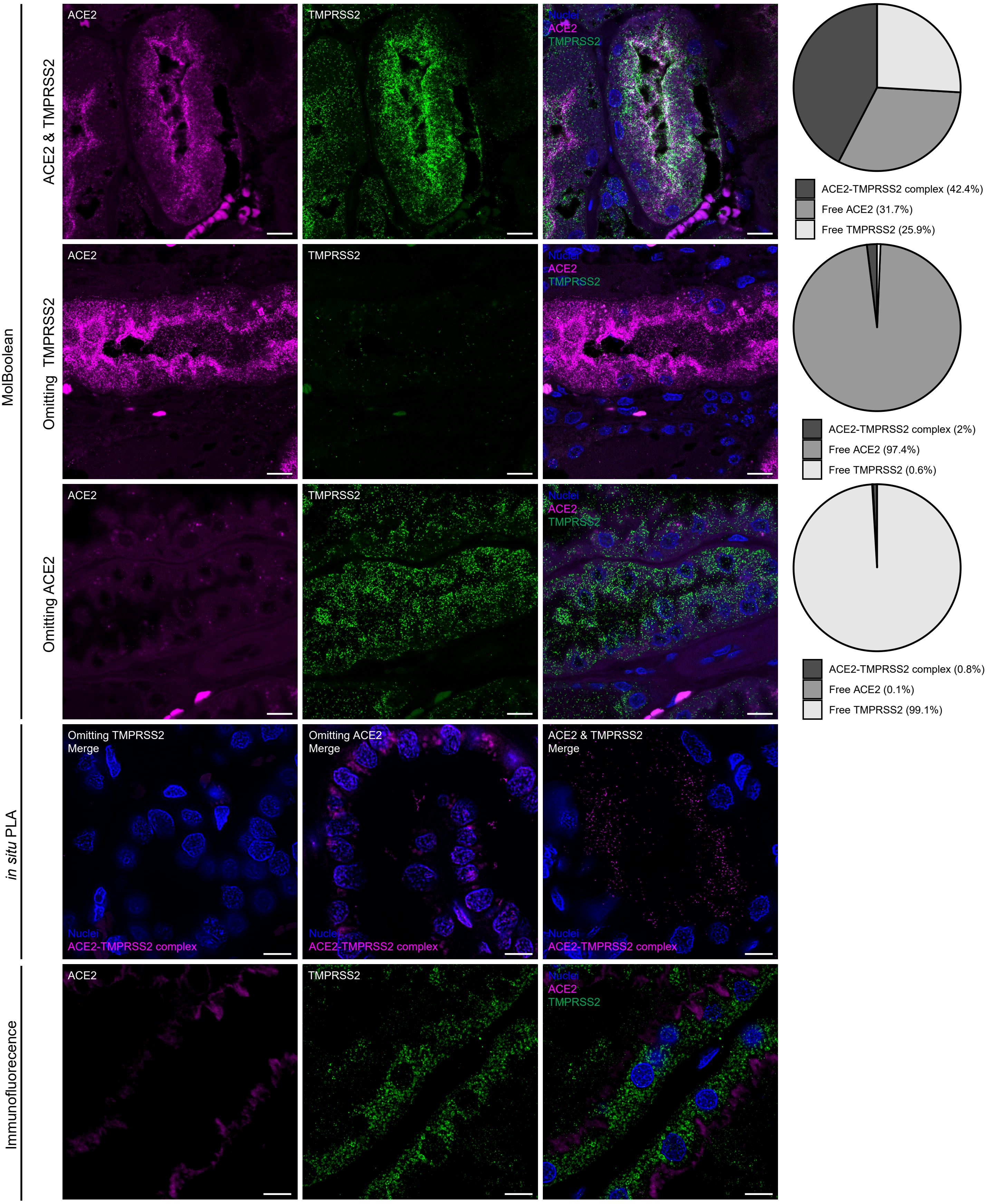

MolBoolean
ACE2 & TMPRSS2
Omitting TMPRSS2
Omitting ACE2
in situ PLA
Immunofluorecence
ACE2
TMPRSS2
Nuclei
ACE2
TMPRSS2
ACE2-TMPRSS2 complex (42.4%)
Free ACE2 (31.7%)
Free TMPRSS2 (25.9%)
ACE2-TMPRSS2 complex (2%)
Free ACE2 (97.4%)
Free TMPRSS2 (0.6%)
ACE2-TMPRSS2 complex (0.8%)
Free ACE2 (0.1%)
Free TMPRSS2 (99.1%)
Omitting TMPRSS2
Merge
Omitting ACE2
Merge
ACE2 & TMPRSS2
Merge
Nuclei
ACE2-TMPRSS2 complex

b.

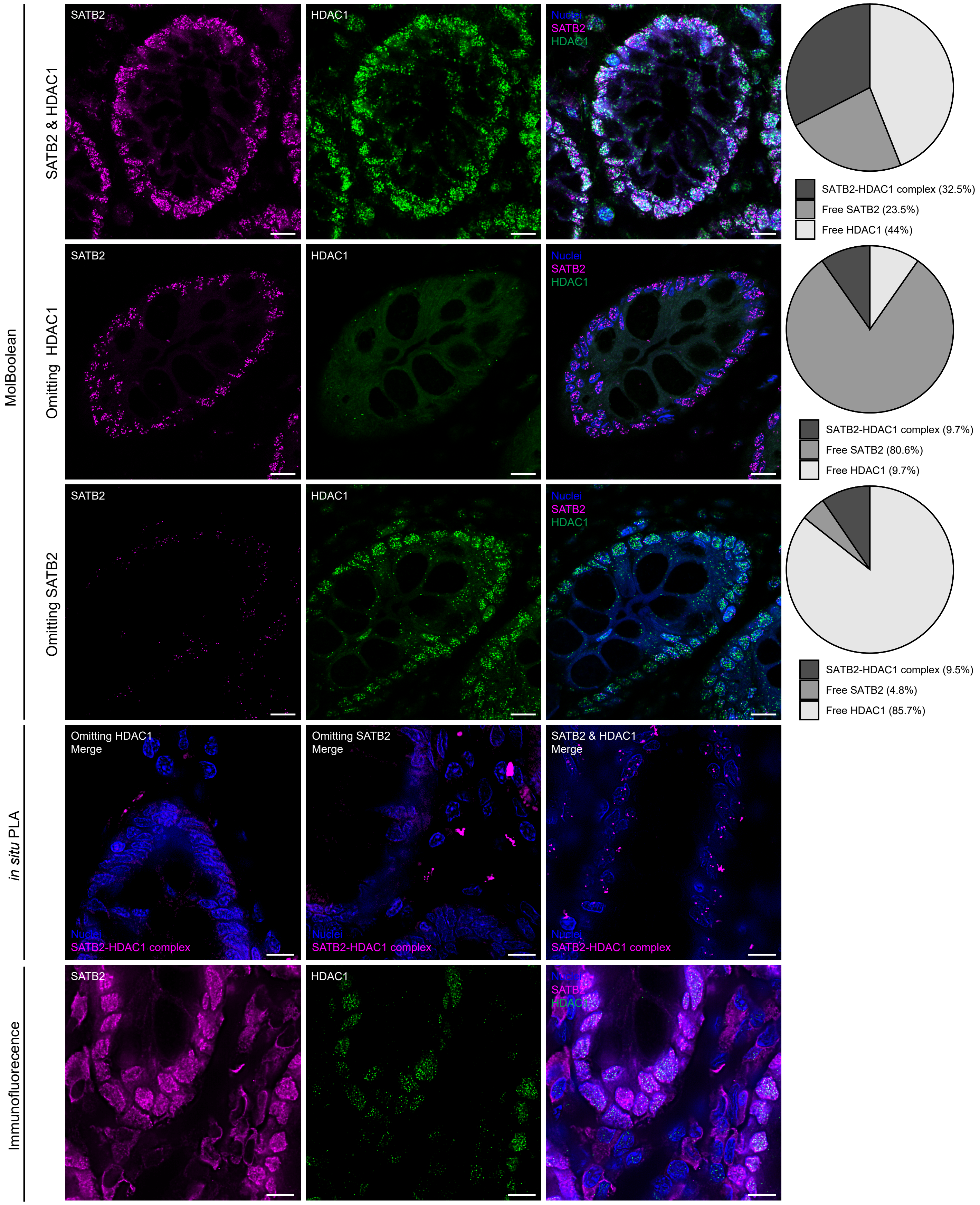
MolBoolean
SATB2 & HDAC1
Omitting HDAC1
Omitting SATB2
SATB2
HDAC1
Nuclei
SATB2-HDAC1 complex (32.5%)
Free SATB2 (23.5%)
Free HDAC1 (44%)
SATB2-HDAC1 complex (9.7%)
Free SATB2 (80.6%)
Free HDAC1 (9.7%)
SATB2-HDAC1 complex (9.5%)
Free SATB2 (4.8%)
Free HDAC1 (85.7%)
in situ PLA
Omitting HDAC1
Merge
Omitting SATB2
SATB2 & HDAC1
SATB2-HDAC1 complex
Immunofluorecence

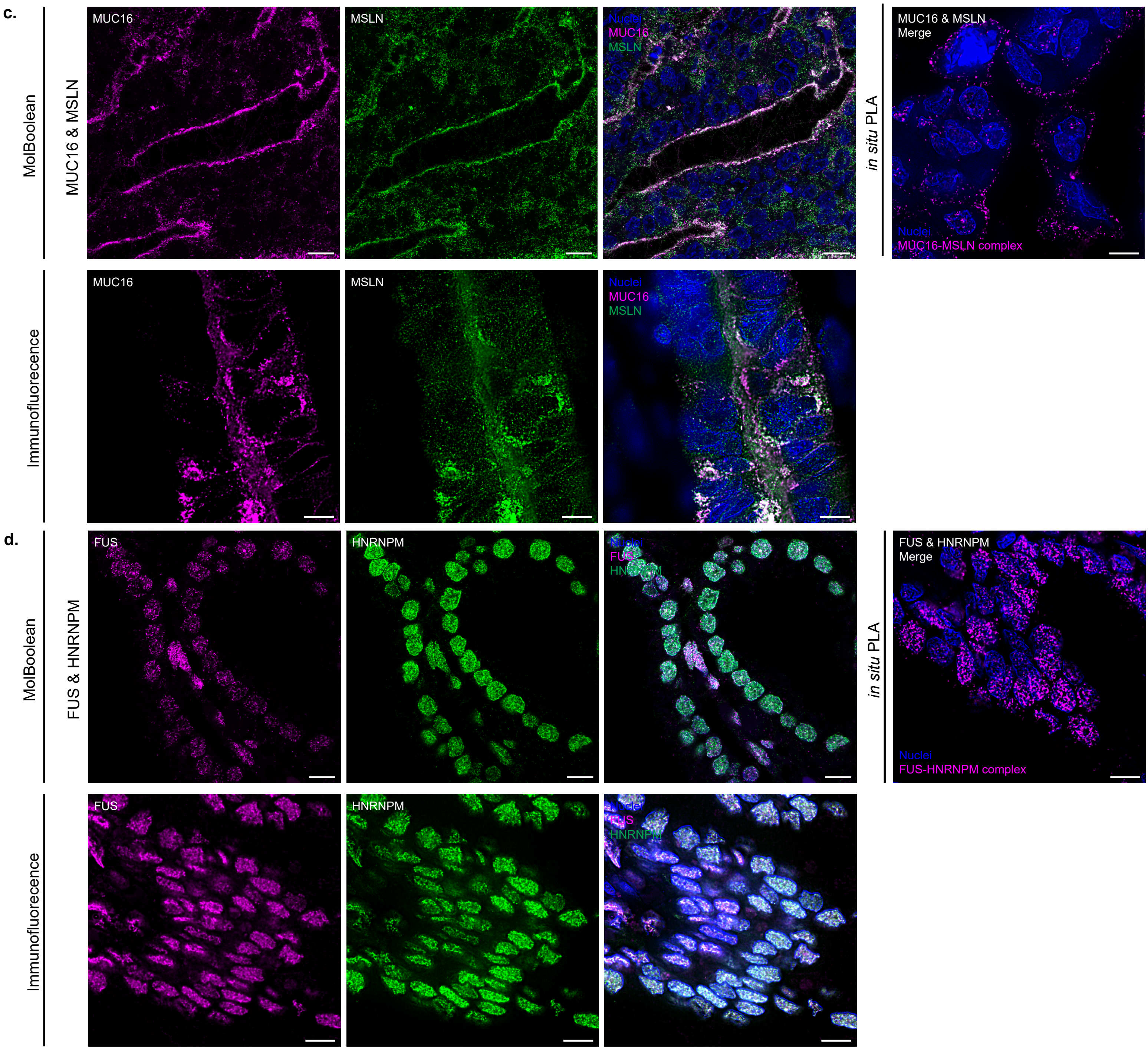

Supplementary Fig. 6 MolBoolean and *in situ* PLA staining and quantification in FFPE tissue sections (extended view of Fig. 8, omitting controls and, IF validation of FFPE tissue co-stains, and additional examples of MolBoolean performance in FFPE tissue sections). (a) ACE2 and TMPRSS2 co-stain in kidney tissue sections. MolBoolean and IF signals for ACE2 shown in magenta, TMPRSS2 in green, ACE2-TMPRSS2 complex in white, and nuclei in blue. *in situ* PLA signals for ACE2-TMPRSS2 complex are shown in magenta, and nuclei in blue. **(b)** SATB2 and HDAC1 co-stain in colon tissue sections. MolBoolean and IF signals for SATB2 shown in magenta, HDAC1 in green, SATB1-HDAC1 complex in white, and nuclei in blue. *in situ* PLA signals for SATB2-HDAC1 complex are shown in magenta, and nuclei in blue. **(c)** MUC16 and MSLN co-stain in ovarian cancer tissue sections. MolBoolean and IF signals for MUC16 shown in magenta, MSLN in green, MUC16-MSLN complex in white, and nuclei in blue. *in situ* PLA signals for MUC16-MSLN complex are shown in magenta, and nuclei in blue.**(d)** FUS and HNRNPM co-stain in prostate tissue sections. MolBoolean and IF signals for FUS shown in magenta, HNRNPM in green, FUS-HNRNPM complex in white, and nuclei in blue. *in situ* PLA signals for FUS-HNRNPM complex are shown in magenta, and nuclei in blue. MolBoolean quantification shown in percentage of RCPs in each category (free protein A, free protein B and, AB complex) per frame. Scale bars = 10µm. Data collected from three independent experiments. Source data are provided as a Source Data file.

5. CellProfiler pipeline for MolBoolean analysis

CellProfiler Pipeline: http://www.cellprofiler.org

Version:4.1.3

DateRevision:421

GitHash:

ModuleCount:44

HasImagePlaneDetails:False

Images:[module_num:1|svn_version:'Unknown'|variable_revision_number:2|show_window:False|notes:['To begin creating your project, use the Images module to compile a list of files and/or folders that you want to analyze. You can also specify a set of rules to include only the desired files in your selected folders.']|batch_state:array([], dtype=uint8)|enabled:True|wants_pause:False]

:

Filter images?:Custom

Select the rule criteria:and (file doesnot contain "Thumbs")

Metadata:[module_num:2|svn_version:'Unknown'|variable_revision_number:6|show_window:False|notes:['The Metadata module optionally allows you to extract information describing your images (i.e, metadata) which will be stored along with your measurements. This information can be contained in the file name and/or location, or in an external file.']|batch_state:array([], dtype=uint8)|enabled:True|wants_pause:False]

Extract metadata?:Yes

Metadata data type:Text

Metadata types:{}

Extraction method count:1

Metadata extraction method:Extract from file/folder names

Metadata source:File name

Regular expression to extract from file name:(?P<Exp>[A-Z]{2}[0-9]{4})_Slide-(?P<Slide>[0-9]{1})_Well_(?P<Well>[A-Z]{1}[0-9]{1})_Image(?P<Number>[0-9]{1,2})_decon_RAW_ch(?P<channel>[0-9]{2})

Regular expression to extract from folder name:(?P<Date>[0-9]{4}_[0-9]{2}_[0-9]{2})$

Extract metadata from:All images

Select the filtering criteria:and (file does contain "")

Metadata file location:Elsewhere...|

Match file and image metadata:[]

Use case insensitive matching?:No

Metadata file name:

Does cached metadata exist?:No

NamesAndTypes:[module_num:3|svn_version:'Unknown'|variable_revision_number:8|show_window:False|notes:['The NamesAndTypes module allows you to assign a meaningful name to each image by which other modules will refer to it.']|batch_state:array([], dtype=uint8)|enabled:True|wants_pause:False]

Assign a name to:Images matching rules

Select the image type:Grayscale image

Name to assign these images:image

Match metadata:[]

Image set matching method:Order

Set intensity range from:Image metadata

Assignments count:3

Single images count:0

Maximum intensity:255.0

Process as 3D?:No

Relative pixel spacing in X:1.0

Relative pixel spacing in Y:1.0

Relative pixel spacing in Z:1.0

Select the rule criteria:and (file does contain "_ch00")

Name to assign these images:DNA

Name to assign these objects:Cell

Select the image type:Grayscale image

Set intensity range from:Image metadata

Maximum intensity:255.0

Select the rule criteria:and (file does contain "_ch01")

Name to assign these images:TexRed

Name to assign these objects:Nucleus

Select the image type:Grayscale image

Set intensity range from:Image metadata

Maximum intensity:255.0

Select the rule criteria:and (file does contain "_ch02")

Name to assign these images:Atto647

Name to assign these objects:Cytoplasm

Select the image type:Grayscale image

Set intensity range from:Image metadata

Maximum intensity:255.0

Groups:[module_num:4|svn_version:'Unknown'|variable_revision_number:2|show_window:False|notes:['The Groups module optionally allows you to split your list of images into image subsets (groups) which will be processed independently of each other. Examples of groupings include screening batches, microtiter plates, time-lapse movies, etc.']|batch_state:array([], dtype=uint8)|enabled:True|wants_pause:False]

Do you want to group your images?:No

grouping metadata count:1

Metadata category:Well

IdentifyPrimaryObjects:[module_num:5|svn_version:'Unknown'|variable_revision_number:15|show_window:False|notes:[]|batch_state:array([], dtype=uint8)|enabled:True|wants_pause:False]

Select the input image:DNA

Name the primary objects to be identified:Nuclei

Typical diameter of objects, in pixel units (Min,Max):300,550

Discard objects outside the diameter range?:Yes

Discard objects touching the border of the image?:Yes

Method to distinguish clumped objects:Shape

Method to draw dividing lines between clumped objects:Shape

Size of smoothing filter:20

Suppress local maxima that are closer than this minimum allowed distance:20.0

Speed up by using lower-resolution image to find local maxima?:Yes

Fill holes in identified objects?:After both thresholding and declumping

Automatically calculate size of smoothing filter for declumping?:Yes

Automatically calculate minimum allowed distance between local maxima?:No

Handling of objects if excessive number of objects identified:Continue

Maximum number of objects:500

Use advanced settings?:Yes

Threshold setting version:12

Threshold strategy:Global

Thresholding method:Otsu

Threshold smoothing scale:1

Threshold correction factor:1

Lower and upper bounds on threshold:0, 1

Manual threshold:0.34

Select the measurement to threshold with:None

Two-class or three-class thresholding?:Two classes

Log transform before thresholding?:No

Assign pixels in the middle intensity class to the foreground or the background?:Foreground

Size of adaptive window:10

Lower outlier fraction:0.05

Upper outlier fraction:0.05

Averaging method:Mean

Variance method:Standard deviation

# of deviations:2

Thresholding method:Default

IdentifySecondaryObjects:[module_num:6|svn_version:'Unknown'|variable_revision_number:10|show_window:False|notes:[]|batch_state:array([], dtype=uint8)|enabled:True|wants_pause:False]

Select the input objects:Nuclei

Name the objects to be identified:Cells

Select the method to identify the secondary objects:Distance - N

Select the input image:TexRed

Number of pixels by which to expand the primary objects:200

Regularization factor:0.05

Discard secondary objects touching the border of the image?:No

Discard the associated primary objects?:No

Name the new primary objects:FilteredNuclei

Fill holes in identified objects?:Yes

Threshold setting version:12

Threshold strategy:Global

Thresholding method:Minimum Cross-Entropy

Threshold smoothing scale:1.3488

Threshold correction factor:1

Lower and upper bounds on threshold:(0.0, 1.0)

Manual threshold:0.0

Select the measurement to threshold with:None

Two-class or three-class thresholding?:Two classes

Log transform before thresholding?:No

Assign pixels in the middle intensity class to the foreground or the background?:Foreground

Size of adaptive window:10

Lower outlier fraction:0.05

Upper outlier fraction:0.05

Averaging method:Mean

Variance method:Standard deviation

# of deviations:2

Thresholding method:Default

EnhanceOrSuppressFeatures:[module_num:7|svn_version:'Unknown'|variable_revision_number:7|show_window:False|notes:[]|batch_state:array([], dtype=uint8)|enabled:True|wants_pause:False]

Select the input image:TexRed

Name the output image:FilteredTexRed

Select the operation:Enhance

Feature size:30

Feature type:Speckles

Range of hole sizes:1,10

Smoothing scale:2.0

Shear angle:0.0

Decay:0.95

Enhancement method:Tubeness

Speed and accuracy:Slow

Rescale result image:Yes

EnhanceOrSuppressFeatures:[module_num:8|svn_version:'Unknown'|variable_revision_number:7|show_window:False|notes:[]|batch_state:array([], dtype=uint8)|enabled:True|wants_pause:False]

Select the input image:Atto647

Name the output image:FilteredAtto647

Select the operation:Enhance

Feature size:30

Feature type:Speckles

Range of hole sizes:1,10

Smoothing scale:2.0

Shear angle:0.0

Decay:0.95

Enhancement method:Tubeness

Speed and accuracy:Slow

Rescale result image:Yes

IdentifyPrimaryObjects:[module_num:9|svn_version:'Unknown'|variable_revision_number:15|show_window:False|notes:[]|batch_state:array([], dtype=uint8)|enabled:True|wants_pause:False]

Select the input image:FilteredAtto647

Name the primary objects to be identified:Atto647_identifyPrimary

Typical diameter of objects, in pixel units (Min,Max):2,25

Discard objects outside the diameter range?:Yes

Discard objects touching the border of the image?:Yes

Method to distinguish clumped objects:Intensity

Method to draw dividing lines between clumped objects:Intensity

Size of smoothing filter:0

Suppress local maxima that are closer than this minimum allowed distance:0

Speed up by using lower-resolution image to find local maxima?:No

Fill holes in identified objects?:Never

Automatically calculate size of smoothing filter for declumping?:No

Automatically calculate minimum allowed distance between local maxima?:No

Handling of objects if excessive number of objects identified:Continue

Maximum number of objects:500

Use advanced settings?:Yes

Threshold setting version:12

Threshold strategy:Global

Thresholding method:Manual

Threshold smoothing scale:1.3488

Threshold correction factor:1.0

Lower and upper bounds on threshold:0.0,1.0

Manual threshold:0.05

Select the measurement to threshold with:None

Two-class or three-class thresholding?:Two classes

Log transform before thresholding?:No

Assign pixels in the middle intensity class to the foreground or the background?:Foreground

Size of adaptive window:10

Lower outlier fraction:0.05

Upper outlier fraction:0.05

Averaging method:Mean

Variance method:Standard deviation

# of deviations:2

Thresholding method:Default

IdentifyPrimaryObjects:[module_num:10|svn_version:'Unknown'|variable_revision_number:15|show_window:False|notes:[]|batch_state:array([], dtype=uint8)|enabled:True|wants_pause:False]

Select the input image:FilteredTexRed

Name the primary objects to be identified:TexRed_identifyPrimary

Typical diameter of objects, in pixel units (Min,Max):2,25

Discard objects outside the diameter range?:Yes

Discard objects touching the border of the image?:Yes

Method to distinguish clumped objects:Intensity

Method to draw dividing lines between clumped objects:Intensity

Size of smoothing filter:0

Suppress local maxima that are closer than this minimum allowed distance:0

Speed up by using lower-resolution image to find local maxima?:No

Fill holes in identified objects?:Never

Automatically calculate size of smoothing filter for declumping?:No

Automatically calculate minimum allowed distance between local maxima?:No

Handling of objects if excessive number of objects identified:Continue

Maximum number of objects:500

Use advanced settings?:Yes

Threshold setting version:12

Threshold strategy:Global

Thresholding method:Manual

Threshold smoothing scale:1.3488

Threshold correction factor:1.0

Lower and upper bounds on threshold:0.0,1.0

Manual threshold:0.05

Select the measurement to threshold with:None

Two-class or three-class thresholding?:Two classes

Log transform before thresholding?:No

Assign pixels in the middle intensity class to the foreground or the background?:Foreground

Size of adaptive window:10

Lower outlier fraction:0.05

Upper outlier fraction:0.05

Averaging method:Mean

Variance method:Standard deviation

# of deviations:2

Thresholding method:Default

ExpandOrShrinkObjects:[module_num:11|svn_version:'Unknown'|variable_revision_number:2|show_window:False|notes:[]|batch_state:array([], dtype=uint8)|enabled:True|wants_pause:False]

Select the input objects:Atto647_identifyPrimary

Name the output objects:Atto647_identifyPrimary_asPoint

Select the operation:Shrink objects to a point

Number of pixels by which to expand or shrink:1

Fill holes in objects so that all objects shrink to a single point?:No

ExpandOrShrinkObjects:[module_num:12|svn_version:'Unknown'|variable_revision_number:2|show_window:False|notes:[]|batch_state:array([], dtype=uint8)|enabled:True|wants_pause:False]

Select the input objects:TexRed_identifyPrimary

Name the output objects:TexRed_identifyPrimary_asPoint

Select the operation:Shrink objects to a point

Number of pixels by which to expand or shrink:1

Fill holes in objects so that all objects shrink to a single point?:No

IdentifySecondaryObjects:[module_num:13|svn_version:'Unknown'|variable_revision_number:10|show_window:False|notes:[]|batch_state:array([], dtype=uint8)|enabled:True|wants_pause:False]

Select the input objects:Atto647_identifyPrimary_asPoint

Name the objects to be identified:Atto647_expanded

Select the method to identify the secondary objects:Distance - B

Select the input image:FilteredAtto647

Number of pixels by which to expand the primary objects:3

Regularization factor:0.05

Discard secondary objects touching the border of the image?:No

Discard the associated primary objects?:No

Name the new primary objects:FilteredNuclei

Fill holes in identified objects?:Yes

Threshold setting version:12

Threshold strategy:Global

Thresholding method:Minimum Cross-Entropy

Threshold smoothing scale:0.0

Threshold correction factor:2.0

Lower and upper bounds on threshold:0.0,1.0

Manual threshold:0.0

Select the measurement to threshold with:None

Two-class or three-class thresholding?:Two classes

Log transform before thresholding?:No

Assign pixels in the middle intensity class to the foreground or the background?:Foreground

Size of adaptive window:50

Lower outlier fraction:0.05

Upper outlier fraction:0.05

Averaging method:Mean

Variance method:Standard deviation

# of deviations:2.0

Thresholding method:Minimum Cross-Entropy

IdentifySecondaryObjects:[module_num:14|svn_version:'Unknown'|variable_revision_number:10|show_window:False|notes:[]|batch_state:array([], dtype=uint8)|enabled:True|wants_pause:False]

Select the input objects:TexRed_identifyPrimary_asPoint

Name the objects to be identified:TexRed_expanded

Select the method to identify the secondary objects:Distance - B

Select the input image:FilteredTexRed

Number of pixels by which to expand the primary objects:3

Regularization factor:0.05

Discard secondary objects touching the border of the image?:No

Discard the associated primary objects?:No

Name the new primary objects:FilteredNuclei

Fill holes in identified objects?:Yes

Threshold setting version:12

Threshold strategy:Global

Thresholding method:Minimum Cross-Entropy

Threshold smoothing scale:0.0

Threshold correction factor:2.0

Lower and upper bounds on threshold:0.0,1.0

Manual threshold:0.0

Select the measurement to threshold with:None

Two-class or three-class thresholding?:Two classes

Log transform before thresholding?:No

Assign pixels in the middle intensity class to the foreground or the background?:Foreground

Size of adaptive window:50

Lower outlier fraction:0.05

Upper outlier fraction:0.05

Averaging method:Mean

Variance method:Standard deviation

# of deviations:2.0

Thresholding method:Minimum Cross-Entropy

CombineObjects:[module_num:15|svn_version:'Unknown'|variable_revision_number:1|show_window:False|notes:[]|batch_state:array([], dtype=uint8)|enabled:True|wants_pause:False]

Select initial object set:Atto647_expanded

Select object set to combine:TexRed_expanded

Select how to handle overlapping objects:Merge

Name the combined object set:AllBlobs

MaskObjects:[module_num:16|svn_version:'Unknown'|variable_revision_number:3|show_window:False|notes:[]|batch_state:array([], dtype=uint8)|enabled:True|wants_pause:False]

Select objects to be masked:AllBlobs

Name the masked objects:BlobsInCells

Mask using a region defined by other objects or by binary image?:Objects

Select the masking object:Cells

Select the masking image:BinaryCy5

Handling of objects that are partially masked:Keep

Fraction of object that must overlap:0.5

Numbering of resulting objects:Renumber

Invert the mask?:No

MeasureObjectIntensity:[module_num:17|svn_version:'Unknown'|variable_revision_number:4|show_window:False|notes:[]|batch_state:array([], dtype=uint8)|enabled:True|wants_pause:False]

Select images to measure:FilteredTexRed, FilteredAtto647

Select objects to measure:BlobsInCells

DisplayDensityPlot:[module_num:18|svn_version:'Unknown'|variable_revision_number:1|show_window:False|notes:[]|batch_state:array([], dtype=uint8)|enabled:True|wants_pause:False]

Select the object to display on the X-axis:BlobsInCells

Select the object measurement to plot on the X-axis:Intensity_MaxIntensity_FilteredTexRed

Select the object to display on the Y-axis:BlobsInCells

Select the object measurement to plot on the Y-axis:Intensity_MaxIntensity_FilteredAtto647

Select the grid size:100

How should the X-axis be scaled?:linear

How should the Y-axis be scaled?:linear

How should the colorbar be scaled?:log

Select the color map:coolwarm

Enter a title for the plot, if desired:Max intensity per blob, x=TexRed, y=Atto647

ClassifyObjects:[module_num:19|svn_version:'Unknown'|variable_revision_number:3|show_window:False|notes:[]|batch_state:array([], dtype=uint8)|enabled:True|wants_pause:False]

Make classification decision based on:Pair of measurements

Hidden:1

Hidden:1

Select the object to be classified:None

Select the measurement to classify by:None

Select bin spacing:Evenly spaced bins

Number of bins:3

Lower threshold:0.0

Use a bin for objects below the threshold?:No

Upper threshold:1.0

Use a bin for objects above the threshold?:No

Enter the custom thresholds separating the values between bins:0,1

Give each bin a name?:No

Enter the bin names separated by commas:None

Retain an image of the classified objects?:No

Name the output image:ClassifiedNuclei

Select a class:None

Name the output objects:ClassifiedObjects

Select the object name:BlobsInCells

Select the first measurement:Intensity_MaxIntensity_FilteredTexRed

Method to select the cutoff:Custom

Enter the cutoff value:0

Select the second measurement:Intensity_MaxIntensity_FilteredAtto647

Method to select the cutoff:Custom

Enter the cutoff value:0

Use custom names for the bins?:Yes

Enter the low-low bin name:background

Enter the low-high bin name:Atto647

Enter the high-low bin name:TexRed

Enter the high-high bin name:both

Retain an image of the classified objects?:Yes

Enter the image name:Classified_blobs

Save classes as new object sets?:No

Select the location of the classifier model file:Default Output Folder|None

Rules or classifier file name:None

FilterObjects:[module_num:20|svn_version:'Unknown'|variable_revision_number:9|show_window:False|notes:[]|batch_state:array([], dtype=uint8)|enabled:True|wants_pause:False]

Select the objects to filter:BlobsInCells

Name the output objects:TexRed_Cells

Select the filtering mode:Measurements

Select the filtering method:Limits

Select the objects that contain the filtered objects:None

Select the location of the rules or classifier file:Elsewhere...|

Rules or classifier file name:rules.txt

Class number:1

Measurement count:1

Additional object count:0

Assign overlapping child to:Both parents

Keep removed objects as a seperate set?:No

Name the objects removed by the filter:RemovedObjects

Select the measurement to filter by:Classify_TexRed

Filter using a minimum measurement value?:Yes

Minimum value:1

Filter using a maximum measurement value?:No

Maximum value:1.0

FilterObjects:[module_num:21|svn_version:'Unknown'|variable_revision_number:9|show_window:False|notes:[]|batch_state:array([], dtype=uint8)|enabled:True|wants_pause:False]

Select the objects to filter:BlobsInCells

Name the output objects:Atto647_Cells

Select the filtering mode:Measurements

Select the filtering method:Limits

Select the objects that contain the filtered objects:None

Select the location of the rules or classifier file:Elsewhere...|

Rules or classifier file name:rules.txt

Class number:1

Measurement count:1

Additional object count:0

Assign overlapping child to:Both parents

Keep removed objects as a seperate set?:No

Name the objects removed by the filter:RemovedObjects

Select the measurement to filter by:Classify_Atto647

Filter using a minimum measurement value?:Yes

Minimum value:1.0

Filter using a maximum measurement value?:No

Maximum value:1.0

FilterObjects:[module_num:22|svn_version:'Unknown'|variable_revision_number:9|show_window:False|notes:[]|batch_state:array([], dtype=uint8)|enabled:True|wants_pause:False]

Select the objects to filter:BlobsInCells

Name the output objects:background_Cells

Select the filtering mode:Measurements

Select the filtering method:Limits

Select the objects that contain the filtered objects:None

Select the location of the rules or classifier file:Elsewhere...|

Rules or classifier file name:rules.txt

Class number:1

Measurement count:1

Additional object count:0

Assign overlapping child to:Both parents

Keep removed objects as a seperate set?:No

Name the objects removed by the filter:RemovedObjects

Select the measurement to filter by:Classify_background

Filter using a minimum measurement value?:Yes

Minimum value:1.0

Filter using a maximum measurement value?:No

Maximum value:1.0

FilterObjects:[module_num:23|svn_version:'Unknown'|variable_revision_number:9|show_window:False|notes:[]|batch_state:array([], dtype=uint8)|enabled:True|wants_pause:False]

Select the objects to filter:BlobsInCells

Name the output objects:Both_Cells

Select the filtering mode:Measurements

Select the filtering method:Limits

Select the objects that contain the filtered objects:None

Select the location of the rules or classifier file:Elsewhere...|

Rules or classifier file name:rules.txt

Class number:1

Measurement count:1

Additional object count:0

Assign overlapping child to:Both parents

Keep removed objects as a seperate set?:No

Name the objects removed by the filter:RemovedObjects

Select the measurement to filter by:Classify_both

Filter using a minimum measurement value?:Yes

Minimum value:1.0

Filter using a maximum measurement value?:No

Maximum value:1.0

OverlayOutlines:[module_num:24|svn_version:'Unknown'|variable_revision_number:4|show_window:False|notes:[]|batch_state:array([], dtype=uint8)|enabled:True|wants_pause:False]

Display outlines on a blank image?:No

Select image on which to display outlines:Classified_blobs

Name the output image:OrigOverlay

Outline display mode:Color

Select method to determine brightness of outlines:Max of image

How to outline:Inner

Select outline color:#40FF40

Select objects to display:Nuclei

SaveImages:[module_num:25|svn_version:'Unknown'|variable_revision_number:16|show_window:False|notes:[]|batch_state:array([], dtype=uint8)|enabled:True|wants_pause:False]

Select the type of image to save:Image

Select the image to save:Classified_blobs

Select method for constructing file names:From image filename

Select image name for file prefix:DNA

Enter single file name:OrigBlue

Number of digits:4

Append a suffix to the image file name?:Yes

Text to append to the image name:_classified_blobs

Saved file format:png

Output file location:Default Output

Image bit depth:8-bit integer

Overwrite existing files without warning?:No

When to save:Every cycle

Record the file and path information to the saved image?:No

Create subfolders in the output folder?:No

Base image folder:Elsewhere...|

How to save the series:T (Time)

Save with lossless compression?:No

SaveImages:[module_num:26|svn_version:'Unknown'|variable_revision_number:16|show_window:False|notes:[]|batch_state:array([], dtype=uint8)|enabled:True|wants_pause:False]

Select the type of image to save:Image

Select the image to save:OrigOverlay

Select method for constructing file names:From image filename

Select image name for file prefix:DNA

Enter single file name:OrigBlue

Number of digits:4

Append a suffix to the image file name?:Yes

Text to append to the image name:_OrigOverlay

Saved file format:png

Output file location:Default Output

Image bit depth:8-bit integer

Overwrite existing files without warning?:No

When to save:Every cycle

Record the file and path information to the saved image?:No

Create subfolders in the output folder?:No

Base image folder:Elsewhere...|

How to save the series:T (Time)

Save with lossless compression?:No

MeasureImageAreaOccupied:[module_num:27|svn_version:'Unknown'|variable_revision_number:5|show_window:False|notes:[]|batch_state:array([], dtype=uint8)|enabled:True|wants_pause:False]

Measure the area occupied by:Objects

Select binary images to measure:

Select object sets to measure:Nuclei

RelateObjects:[module_num:28|svn_version:'Unknown'|variable_revision_number:5|show_window:False|notes:[]|batch_state:array([], dtype=uint8)|enabled:True|wants_pause:False]

Parent objects:Cells

Child objects:TexRed_Cells

Calculate child-parent distances?:None

Calculate per-parent means for all child measurements?:Yes

Calculate distances to other parents?:No

Do you want to save the children with parents as a new object set?:Yes

Name the output object:RelateObjects_TxR

Parent name:None

Parent name:None

Parent name:None

Parent name:None

Parent name:None

Parent name:None

Parent name:None

Parent name:None

RelateObjects:[module_num:29|svn_version:'Unknown'|variable_revision_number:5|show_window:False|notes:[]|batch_state:array([], dtype=uint8)|enabled:True|wants_pause:False]

Parent objects:Cells

Child objects:Atto647_Cells

Calculate child-parent distances?:None

Calculate per-parent means for all child measurements?:Yes

Calculate distances to other parents?:No

Do you want to save the children with parents as a new object set?:Yes

Name the output object:RelateObjects_Atto647

Parent name:None

Parent name:None

Parent name:None

Parent name:None

Parent name:None

Parent name:None

Parent name:None

Parent name:None

RelateObjects:[module_num:30|svn_version:'Unknown'|variable_revision_number:5|show_window:False|notes:[]|batch_state:array([], dtype=uint8)|enabled:True|wants_pause:False]

Parent objects:Cells

Child objects:Both_Cells

Calculate child-parent distances?:None

Calculate per-parent means for all child measurements?:Yes

Calculate distances to other parents?:No

Do you want to save the children with parents as a new object set?:Yes

Name the output object:RelateObjects_Both

Parent name:None

Parent name:None

Parent name:None

Parent name:None

Parent name:None

Parent name:None

Parent name:None

Parent name:None

RelateObjects:[module_num:31|svn_version:'Unknown'|variable_revision_number:5|show_window:False|notes:[]|batch_state:array([], dtype=uint8)|enabled:True|wants_pause:False]

Parent objects:Cells

Child objects:background_Cells

Calculate child-parent distances?:None

Calculate per-parent means for all child measurements?:Yes

Calculate distances to other parents?:No

Do you want to save the children with parents as a new object set?:Yes

Name the output object:RelateObjects_Background

Parent name:None

Parent name:None

Parent name:None

Parent name:None

Parent name:None

Parent name:None

Parent name:None

Parent name:None

ExpandOrShrinkObjects:[module_num:32|svn_version:'Unknown'|variable_revision_number:2|show_window:False|notes:[]|batch_state:array([], dtype=uint8)|enabled:True|wants_pause:False]

Select the input objects:Atto647_Cells

Name the output objects:Atto647_Cell_asPoints

Select the operation:Shrink objects to a point

Number of pixels by which to expand or shrink:1

Fill holes in objects so that all objects shrink to a single point?:No

ExpandOrShrinkObjects:[module_num:33|svn_version:'Unknown'|variable_revision_number:2|show_window:False|notes:[]|batch_state:array([], dtype=uint8)|enabled:True|wants_pause:False]

Select the input objects:TexRed_Cells

Name the output objects:TexRed_Cells_asPoints

Select the operation:Shrink objects to a point

Number of pixels by which to expand or shrink:1

Fill holes in objects so that all objects shrink to a single point?:No

ExpandOrShrinkObjects:[module_num:34|svn_version:'Unknown'|variable_revision_number:2|show_window:False|notes:[]|batch_state:array([], dtype=uint8)|enabled:True|wants_pause:False]

Select the input objects:Both_Cells

Name the output objects:Both_Cells_asPoints

Select the operation:Shrink objects to a point

Number of pixels by which to expand or shrink:1

Fill holes in objects so that all objects shrink to a single point?:No

ConvertObjectsToImage:[module_num:35|svn_version:'Unknown'|variable_revision_number:1|show_window:False|notes:[]|batch_state:array([], dtype=uint8)|enabled:True|wants_pause:False]

Select the input objects:Atto647_Cell_asPoints

Name the output image:Atto647_asPoints_img

Select the color format:Binary (black & white)

Select the colormap:Default

ConvertObjectsToImage:[module_num:36|svn_version:'Unknown'|variable_revision_number:1|show_window:False|notes:[]|batch_state:array([], dtype=uint8)|enabled:True|wants_pause:False]

Select the input objects:TexRed_Cells_asPoints

Name the output image:TexRed_Cells_asPoints_img

Select the color format:Binary (black & white)

Select the colormap:Default

ConvertObjectsToImage:[module_num:37|svn_version:'Unknown'|variable_revision_number:1|show_window:False|notes:[]|batch_state:array([], dtype=uint8)|enabled:True|wants_pause:False]

Select the input objects:Both_Cells_asPoints

Name the output image:Both_Cells_asPoints_img

Select the color format:Binary (black & white)

Select the colormap:Default

OverlayOutlines:[module_num:38|svn_version:'Unknown'|variable_revision_number:4|show_window:False|notes:[]|batch_state:array([], dtype=uint8)|enabled:True|wants_pause:False]

Display outlines on a blank image?:Yes

Select image on which to display outlines:None

Name the output image:BlobsInCells_img

Outline display mode:Grayscale

Select method to determine brightness of outlines:Max of image

How to outline:Outer

Select outline color:Red

Select objects to display:BlobsInCells

GrayToColor:[module_num:39|svn_version:'Unknown'|variable_revision_number:4|show_window:False|notes:[]|batch_state:array([], dtype=uint8)|enabled:True|wants_pause:False]

Select a color scheme:Stack

Rescale intensity:Yes

Select the image to be colored red:Leave this black

Select the image to be colored green:Leave this black

Select the image to be colored blue:Leave this black

Name the output image:QualityControl_Stack

Relative weight for the red image:1.0

Relative weight for the green image:1.0

Relative weight for the blue image:1.0

Select the image to be colored cyan:Leave this black

Select the image to be colored magenta:Leave this black

Select the image to be colored yellow:Leave this black

Select the image that determines brightness:Leave this black

Relative weight for the cyan image:1.0

Relative weight for the magenta image:1.0

Relative weight for the yellow image:1.0

Relative weight for the brightness image:1.0

Hidden:6

Image name:TexRed

Color:#ff0000

Weight:1.0

Image name:Atto647

Color:#00ff00

Weight:1.0

Image name:TexRed_Cells_asPoints_img

Color:#0000ff

Weight:1.0

Image name:Atto647_asPoints_img

Color:#808000

Weight:1.0

Image name:Both_Cells_asPoints_img

Color:#800080

Weight:1.0

Image name:BlobsInCells_img

Color:#008080

Weight:1.0

GrayToColor:[module_num:40|svn_version:'Unknown'|variable_revision_number:4|show_window:False|notes:[]|batch_state:array([], dtype=uint8)|enabled:True|wants_pause:False]

Select a color scheme:RGB

Rescale intensity:Yes

Select the image to be colored red:TexRed

Select the image to be colored green:Atto647

Select the image to be colored blue:Leave this black

Name the output image:TexRed_Atto647

Relative weight for the red image:1.0

Relative weight for the green image:1.0

Relative weight for the blue image:1.0

Select the image to be colored cyan:Leave this black

Select the image to be colored magenta:Leave this black

Select the image to be colored yellow:Leave this black

Select the image that determines brightness:Leave this black

Relative weight for the cyan image:1.0

Relative weight for the magenta image:1.0

Relative weight for the yellow image:1.0

Relative weight for the brightness image:1.0

Hidden:1

Image name:None

Color:#ff0000

Weight:1.0

OverlayOutlines:[module_num:41|svn_version:'Unknown'|variable_revision_number:4|show_window:False|notes:[]|batch_state:array([], dtype=uint8)|enabled:True|wants_pause:False]

Display outlines on a blank image?:No

Select image on which to display outlines:TexRed_Atto647

Name the output image:QualityControl_SingleImg

Outline display mode:Color

Select method to determine brightness of outlines:Max of image

How to outline:Inner

Select outline color:white

Select objects to display:AllBlobs

Select outline color:magenta

Select objects to display:TexRed_Cells_asPoints

Select outline color:#00FF40

Select objects to display:Atto647_Cell_asPoints

Select outline color:yellow

Select objects to display:Both_Cells_asPoints

SaveImages:[module_num:42|svn_version:'Unknown'|variable_revision_number:16|show_window:False|notes:[]|batch_state:array([], dtype=uint8)|enabled:True|wants_pause:False]

Select the type of image to save:Image

Select the image to save:QualityControl_Stack

Select method for constructing file names:From image filename

Select image name for file prefix:Atto647

Enter single file name:detection_individual

Number of digits:4

Append a suffix to the image file name?:Yes

Text to append to the image name:_QC_stack

Saved file format:tiff

Output file location:Default Output

Image bit depth:8-bit integer

Overwrite existing files without warning?:No

When to save:Every cycle

Record the file and path information to the saved image?:No

Create subfolders in the output folder?:No

Base image folder:Elsewhere...|

How to save the series:T (Time)

Save with lossless compression?:Yes

SaveImages:[module_num:43|svn_version:'Unknown'|variable_revision_number:16|show_window:False|notes:[]|batch_state:array([], dtype=uint8)|enabled:True|wants_pause:False]

Select the type of image to save:Image

Select the image to save:QualityControl_SingleImg

Select method for constructing file names:From image filename

Select image name for file prefix:Atto647

Enter single file name:detection_individual

Number of digits:4

Append a suffix to the image file name?:Yes

Text to append to the image name:_QC_image

Saved file format:tiff

Output file location:Default Output Folder|\\\\argos.rudbeck.uu.se\\MyFolder$\\deske354\\Projects\\MolBoolean\\MolBoolean CS\\Results\\CellProfiler Results\\BX4133_BX4141_EMD-LMNB1\\EMD-LMNB1_all_no classification_22-01-12\\BX4133_BX4141

Image bit depth:8-bit integer

Overwrite existing files without warning?:No

When to save:Every cycle

Record the file and path information to the saved image?:No

Create subfolders in the output folder?:No

Base image folder:Elsewhere...|

How to save the series:T (Time)

Save with lossless compression?:Yes

ExportToSpreadsheet:[module_num:44|svn_version:'Unknown'|variable_revision_number:13|show_window:False|notes:[]|batch_state:array([], dtype=uint8)|enabled:True|wants_pause:True]

Select the column delimiter:Comma (",")

Add image metadata columns to your object data file?:No

Add image file and folder names to your object data file?:No

Select the measurements to export:Yes

Calculate the per-image mean values for object measurements?:No

Calculate the per-image median values for object measurements?:No

Calculate the per-image standard deviation values for object measurements?:No

Output file location:Default Output

Create a GenePattern GCT file?:No

Select source of sample row name:Metadata

Select the image to use as the identifier:None

Select the metadata to use as the identifier:None

Export all measurement types?:Yes

Press button to select measurements:Cells|Location_Center_Z,Cells|Location_Center_X,Cells|Location_Center_Y,Cells|Children_Both_Cells_Count,Cells|Children_Atto647_Cells_Count,Cells|Children_background_Cells_Count,Cells|Children_TexRed_Cells_Count,Cells|Mean_Both_Cells_Location_Center_Y,Cells|Mean_Both_Cells_Location_Center_Z,Cells|Mean_Both_Cells_Location_Center_X,Cells|Mean_Both_Cells_Number_Object_Number,Cells|Mean_background_Cells_Location_Center_X,Cells|Mean_background_Cells_Location_Center_Z,Cells|Mean_background_Cells_Location_Center_Y,Cells|Mean_background_Cells_Number_Object_Number,Cells|Mean_TexRed_Cells_Location_Center_X,Cells|Mean_TexRed_Cells_Location_Center_Z,Cells|Mean_TexRed_Cells_Location_Center_Y,Cells|Mean_TexRed_Cells_Number_Object_Number,Cells|Mean_Atto647_Cells_Number_Object_Number,Cells|Mean_Atto647_Cells_Locat

ion_Center_X,Cells|Mean_Atto647_Cells_Location_Center_Z,Cells|Mean_Atto647_Cells_L
ocation_Center_Y,Cells|Number_Object_Number,Cells|Parent_Nuclei,Atto647_expanded|Nu
mber_Object_Number,Atto647_expanded|Location_Center_Z,Atto647_expanded|Location_C
enter_X,Atto647_expanded|Location_Center_Y,Atto647_expanded|Parent_Atto647_identifyP
rimary_asPoint,Image|ExecutionTime_21FilterObjects,Image|ExecutionTime_29RelateObject
s,Image|ExecutionTime_12ExpandOrShrinkObjects,Image|ExecutionTime_33ExpandOrShrin
kObjects,Image|ExecutionTime_20FilterObjects,Image|ExecutionTime_10IdentifyPrimaryOb
jects,Image|ExecutionTime_34ExpandOrShrinkObjects,Image|ExecutionTime_11ExpandOrS
hrinkObjects,Image|ExecutionTime_18DisplayDensityPlot,Image|ExecutionTime_13Identify
SecondaryObjects,Image|ExecutionTime_27MeasureImageAreaOccupied,Image|ExecutionTi
me_02Metadata,Image|ExecutionTime_38OverlayOutlines,Image|ExecutionTime_14Identify
SecondaryObjects,Image|ExecutionTime_36ConvertObjectsToImage,Image|ExecutionTime_
09IdentifyPrimaryObjects,Image|ExecutionTime_15CombineObjects,Image|ExecutionTime_
17MeasureObjectIntensity,Image|ExecutionTime_30RelateObjects,Image|ExecutionTime_01I
mages,Image|ExecutionTime_43SaveImages,Image|ExecutionTime_35ConvertObjectsToIma
ge,Image|ExecutionTime_25SaveImages,Image|ExecutionTime_04Groups,Image|ExecutionT
ime_23FilterObjects,Image|ExecutionTime_19ClassifyObjects,Image|ExecutionTime_31Rela
teObjects,Image|ExecutionTime_07EnhanceOrSuppressFeatures,Image|ExecutionTime_40Gr
ayToColor,Image|ExecutionTime_41OverlayOutlines,Image|ExecutionTime_16MaskObjects,
Image|ExecutionTime_28RelateObjects,Image|ExecutionTime_03NamesAndTypes,Image|Ex
ecutionTime_39GrayToColor,Image|ExecutionTime_24OverlayOutlines,Image|ExecutionTi
me_37ConvertObjectsToImage,Image|ExecutionTime_08EnhanceOrSuppressFeatures,Image|
ExecutionTime_32ExpandOrShrinkObjects,Image|ExecutionTime_05IdentifyPrimaryObjects
,Image|ExecutionTime_26SaveImages,Image|ExecutionTime_06IdentifySecondaryObjects,I
mage|ExecutionTime_42SaveImages,Image|ExecutionTime_22FilterObjects,Image|Metadata
_Frame,Image|Metadata_Well,Image|Metadata_Exp,Image|Metadata_FileLocation,Image|Met
adata_Slide,Image|Metadata_channel,Image|Metadata_Series,Image|Metadata_Number,Image
|AreaOccupied_Perimeter_Nuclei,Image|AreaOccupied_TotalArea_Nuclei,Image|AreaOccupi
ed_AreaOccupied_Nuclei,Image|Count_BlobsInCells,Image|Count_TexRed_identifyPrimary,
Image|Count_TexRed_identifyPrimary_asPoint,Image|Count_TexRed_Cells,Image|Count_Te
xRed_Cells_asPoints,Image|Count_TexRed_expanded,Image|Count_RelateObjects_Both,Ima
ge|Count_RelateObjects_Atto647,Image|Count_RelateObjects_TxR,Image|Count_RelateObje
cts_Background,Image|Count_Atto647_identifyPrimary,Image|Count_Atto647_identifyPrima
ry_asPoint,Image|Count_Atto647_Cell_asPoints,Image|Count_Atto647_Cells,Image|Count_A
tto647_expanded,Image|Count_Both_Cells,Image|Count_Both_Cells_asPoints,Image|Count_
Nuclei,Image|Count_AllBlobs,Image|Count_background_Cells,Image|Count_Cells,Image|Fra
me_DNA,Image|Frame_TexRed,Image|Frame_Atto647,Image|ModuleError_24OverlayOutlin
es,Image|ModuleError_42SaveImages,Image|ModuleError_35ConvertObjectsToImage,Image
|ModuleError_40GrayToColor,Image|ModuleError_19ClassifyObjects,Image|ModuleError_0
7EnhanceOrSuppressFeatures,Image|ModuleError_27MeasureImageAreaOccupied,Image|Mo
duleError_16MaskObjects,Image|ModuleError_01Images,Image|ModuleError_10IdentifyPri
maryObjects,Image|ModuleError_13IdentifySecondaryObjects,Image|ModuleError_39GrayT
oColor,Image|ModuleError_06IdentifySecondaryObjects,Image|ModuleError_18DisplayDens
ityPlot,Image|ModuleError_43SaveImages,Image|ModuleError_41OverlayOutlines,Image|Mo
duleError_23FilterObjects,Image|ModuleError_20FilterObjects,Image|ModuleError_30Relate
Objects,Image|ModuleError_36ConvertObjectsToImage,Image|ModuleError_17MeasureObje
ctIntensity,Image|ModuleError_21FilterObjects,Image|ModuleError_28RelateObjects,Image|

ModuleError_12ExpandOrShrinkObjects,Image|ModuleError_11ExpandOrShrinkObjects,Im
age|ModuleError_34ExpandOrShrinkObjects,Image|ModuleError_32ExpandOrShrinkObjects
,Image|ModuleError_33ExpandOrShrinkObjects,Image|ModuleError_03NamesAndTypes,Im
age|ModuleError_22FilterObjects,Image|ModuleError_14IdentifySecondaryObjects,Image|M
oduleError_05IdentifyPrimaryObjects,Image|ModuleError_37ConvertObjectsToImage,Image|
ModuleError_08EnhanceOrSuppressFeatures,Image|ModuleError_31RelateObjects,Image|M
oduleError_38OverlayOutlines,Image|ModuleError_25SaveImages,Image|ModuleError_29Re
lateObjects,Image|ModuleError_02Metadata,Image|ModuleError_04Groups,Image|ModuleEr
ror_15CombineObjects,Image|ModuleError_09IdentifyPrimaryObjects,Image|ModuleError_2
6SaveImages,Image|Threshold_WeightedVariance_Nuclei,Image|Threshold_WeightedVarian
ce_Atto647_identifyPrimary,Image|Threshold_WeightedVariance_Atto647_expanded,Image|
Threshold_WeightedVariance_TexRed_expanded,Image|Threshold_WeightedVariance_TexR
ed_identifyPrimary,Image|Threshold_SumOfEntropies_Atto647_expanded,Image|Threshold_
SumOfEntropies_Atto647_identifyPrimary,Image|Threshold_SumOfEntropies_TexRed_ident
ifyPrimary,Image|Threshold_SumOfEntropies_TexRed_expanded,Image|Threshold_SumOfE
ntropies_Nuclei,Image|Threshold_FinalThreshold_Atto647_identifyPrimary,Image|Threshold
_FinalThreshold_Atto647_expanded,Image|Threshold_FinalThreshold_Nuclei,Image|Thresho
ld_FinalThreshold_TexRed_identifyPrimary,Image|Threshold_FinalThreshold_TexRed_expa
nded,Image|Threshold_OrigThreshold_Atto647_expanded,Image|Threshold_OrigThreshold_
Atto647_identifyPrimary,Image|Threshold_OrigThreshold_TexRed_identifyPrimary,Image|T
hreshold_OrigThreshold_TexRed_expanded,Image|Threshold_OrigThreshold_Nuclei,Image|
Series_TexRed,Image|Series_Atto647,Image|Series_DNA,Image|URL_DNA,Image|URL_Att
o647,Image|URL_TexRed,Image|FileName_TexRed,Image|FileName_Atto647,Image|FileNa
me_DNA,Image|Classify_TexRed_PctObjectsPerBin,Image|Classify_TexRed_NumObjectsP
erBin,Image|Classify_background_NumObjectsPerBin,Image|Classify_background_PctObjec
tsPerBin,Image|Classify_both_NumObjectsPerBin,Image|Classify_both_PctObjectsPerBin,Im
age|Classify_Atto647_PctObjectsPerBin,Image|Classify_Atto647_NumObjectsPerBin,Image|
MD5Digest_TexRed,Image|MD5Digest_Atto647,Image|MD5Digest_DNA,Image|Height_DN
A,Image|Height_Atto647,Image|Height_TexRed,Image|Scaling_Atto647,Image|Scaling_Tex
Red,Image|Scaling_DNA,Image|PathName_DNA,Image|PathName_Atto647,Image|PathNam
e_TexRed,Image|Width_Atto647,Image|Width_TexRed,Image|Width_DNA,Image|Group_In
dex,Image|Group_Number,BlobsInCells|Intensity_MaxIntensity_FilteredAtto647,BlobsInCell
s|Intensity_MaxIntensity_FilteredTexRed,BlobsInCells|Intensity_MinIntensity_FilteredTexR
ed,BlobsInCells|Intensity_MinIntensity_FilteredAtto647,BlobsInCells|Intensity_StdIntensityE
dge_FilteredAtto647,BlobsInCells|Intensity_StdIntensityEdge_FilteredTexRed,BlobsInCells|I
ntensity_MinIntensityEdge_FilteredTexRed,BlobsInCells|Intensity_MinIntensityEdge_Filtere
dAtto647,BlobsInCells|Intensity_IntegratedIntensityEdge_FilteredTexRed,BlobsInCells|Inten
sity_IntegratedIntensityEdge_FilteredAtto647,BlobsInCells|Intensity_MaxIntensityEdge_Filt
eredAtto647,BlobsInCells|Intensity_MaxIntensityEdge_FilteredTexRed,BlobsInCells|Intensit
y_UpperQuartileIntensity_FilteredAtto647,BlobsInCells|Intensity_UpperQuartileIntensity_Fil
teredTexRed,BlobsInCells|Intensity_LowerQuartileIntensity_FilteredAtto647,BlobsInCells|In
tensity_LowerQuartileIntensity_FilteredTexRed,BlobsInCells|Intensity_MADIntensity_Filter
edTexRed,BlobsInCells|Intensity_MADIntensity_FilteredAtto647,BlobsInCells|Intensity_Me
dianIntensity_FilteredAtto647,BlobsInCells|Intensity_MedianIntensity_FilteredTexRed,Blobs
InCells|Intensity_MeanIntensity_FilteredTexRed,BlobsInCells|Intensity_MeanIntensity_Filter
edAtto647,BlobsInCells|Intensity_StdIntensity_FilteredAtto647,BlobsInCells|Intensity_StdInt
ensity_FilteredTexRed,BlobsInCells|Intensity_IntegratedIntensity_FilteredTexRed,BlobsInCe

lls|Intensity_IntegratedIntensity_FilteredAtto647,BlobsInCells|Intensity_MeanIntensityEdge_
FilteredTexRed,BlobsInCells|Intensity_MeanIntensityEdge_FilteredAtto647,BlobsInCells|Int
ensity_MassDisplacement_FilteredTexRed,BlobsInCells|Intensity_MassDisplacement_Filtere
dAtto647,BlobsInCells|Classify_Atto647,BlobsInCells|Classify_TexRed,BlobsInCells|Classif
y_background,BlobsInCells|Classify_both,BlobsInCells|Location_MaxIntensity_Y_FilteredA
tto647,BlobsInCells|Location_MaxIntensity_Y_FilteredTexRed,BlobsInCells|Location_MaxI
ntensity_Z_FilteredTexRed,BlobsInCells|Location_MaxIntensity_Z_FilteredAtto647,BlobsIn
Cells|Location_MaxIntensity_X_FilteredTexRed,BlobsInCells|Location_MaxIntensity_X_Fil
teredAtto647,BlobsInCells|Location_CenterMassIntensity_Z_FilteredAtto647,BlobsInCells|L
ocation_CenterMassIntensity_Z_FilteredTexRed,BlobsInCells|Location_CenterMassIntensity
_Y_FilteredAtto647,BlobsInCells|Location_CenterMassIntensity_Y_FilteredTexRed,BlobsIn
Cells|Location_CenterMassIntensity_X_FilteredAtto647,BlobsInCells|Location_CenterMassI
ntensity_X_FilteredTexRed,BlobsInCells|Location_Center_X,BlobsInCells|Location_Center_
Y,BlobsInCells|Children_background_Cells_Count,BlobsInCells|Children_Both_Cells_Count
,BlobsInCells|Children_Atto647_Cells_Count,BlobsInCells|Children_TexRed_Cells_Count,B
lobsInCells|Parent_AllBlobs,BlobsInCells|Number_Object_Number,TexRed_Cells|Parent_Bl
obsInCells,TexRed_Cells|Parent_Cells,TexRed_Cells|Children_RelateObjects_TxR_Count,T
exRed_Cells|Location_Center_Z,TexRed_Cells|Location_Center_Y,TexRed_Cells|Location_
Center_X,TexRed_Cells|Number_Object_Number,Atto647_Cells|Location_Center_Y,Atto64
7_Cells|Location_Center_Z,Atto647_Cells|Location_Center_X,Atto647_Cells|Children_Relat
eObjects_Atto647_Count,Atto647_Cells|Parent_Cells,Atto647_Cells|Parent_BlobsInCells,Att
o647_Cells|Number_Object_Number,TexRed_identifyPrimary|Location_Center_Y,TexRed_i
dentifyPrimary|Location_Center_Z,TexRed_identifyPrimary|Location_Center_X,TexRed_ide
ntifyPrimary|Number_Object_Number,Atto647_identifyPrimary_asPoint|Children_Atto647_e
xpanded_Count,Atto647_identifyPrimary_asPoint|Location_Center_Y,Atto647_identifyPrim
ary_asPoint|Location_Center_X,Atto647_identifyPrimary_asPoint|Number_Object_Number,
Both_Cells|Location_Center_Z,Both_Cells|Location_Center_Y,Both_Cells|Location_Center_
X,Both_Cells|Parent_Cells,Both_Cells|Parent_BlobsInCells,Both_Cells|Children_RelateObje
cts_Both_Count,Both_Cells|Number_Object_Number,RelateObjects_TxR|Number_Object_N
umber,RelateObjects_TxR|Location_Center_Y,RelateObjects_TxR|Location_Center_X,Relat
eObjects_TxR|Location_Center_Z,RelateObjects_TxR|Parent_TexRed_Cells,RelateObjects_
Atto647|Location_Center_X,RelateObjects_Atto647|Location_Center_Y,RelateObjects_Atto
647|Location_Center_Z,RelateObjects_Atto647|Number_Object_Number,RelateObjects_Atto
647|Parent_Atto647_Cells,AllBlobs|Location_Center_X,AllBlobs|Location_Center_Y,AllBlo
bs|Children_BlobsInCells_Count,AllBlobs|Number_Object_Number,Atto647_Cell_asPoints|
Number_Object_Number,Atto647_Cell_asPoints|Location_Center_X,Atto647_Cell_asPoints|
Location_Center_Y,background_Cells|Parent_BlobsInCells,background_Cells|Parent_Cells,b
ackground_Cells|Children_RelateObjects_Background_Count,background_Cells|Number_Ob
ject_Number,background_Cells|Location_Center_Y,background_Cells|Location_Center_Z,ba
ckground_Cells|Location_Center_X,TexRed_identifyPrimary_asPoint|Location_Center_Y,Te
xRed_identifyPrimary_asPoint|Location_Center_X,TexRed_identifyPrimary_asPoint|Childre
n_TexRed_expanded_Count,TexRed_identifyPrimary_asPoint|Number_Object_Number,Tex
Red_expanded|Location_Center_X,TexRed_expanded|Location_Center_Z,TexRed_expanded
|Location_Center_Y,TexRed_expanded|Parent_TexRed_identifyPrimary_asPoint,TexRed_ex
panded|Number_Object_Number,Nuclei|Location_Center_Y,Nuclei|Location_Center_X,Nucl
ei|Number_Object_Number,Nuclei|Children_Cells_Count,Experiment|Pipeline_Pipeline,Expe
riment|Run_Timestamp,Experiment|CellProfiler_Version,Experiment|Modification_Timesta

mp,Both_Cells_asPoints|Location_Center_Y,Both_Cells_asPoints|Location_Center_X,Both_Cells_asPoints|Number_Object_Number,RelateObjects_Both|Location_Center_Y,RelateObjects_Both|Location_Center_Z,RelateObjects_Both|Location_Center_X,RelateObjects_Both|Number_Object_Number,RelateObjects_Both|Parent_Both_Cells,Atto647_identifyPrimary|Number_Object_Number,Atto647_identifyPrimary|Location_Center_Z,Atto647_identifyPrimary|Location_Center_X,Atto647_identifyPrimary|Location_Center_Y,RelateObjects_Background|Location_Center_X,RelateObjects_Background|Location_Center_Y,RelateObjects_Background|Location_Center_Z,RelateObjects_Background|Number_Object_Number,RelateObjects_Background|Parent_background_Cells,TexRed_Cells_asPoints|Location_Center_Y,TexRed_Cells_asPoints|Location_Center_X,TexRed_Cells_asPoints|Number_Object_Number

Representation of Nan/Inf:NaN

Add a prefix to file names?:Yes

Filename prefix:

Overwrite existing files without warning?:No

Data to export:Experiment

Combine these object measurements with those of the previous object?:No

File name:DATA.csv

Use the object name for the file name?:Yes

Data to export:Image

Combine these object measurements with those of the previous object?:No

File name:DATA.csv

Use the object name for the file name?:Yes

6. CellProfiler pipeline for in situ PLA analysis

CellProfiler Pipeline: http://www.cellprofiler.org

Version:4.1.3

DateRevision:421

GitHash:

ModuleCount:11

HasImagePlaneDetails:False

Images:[module_num:1|svn_version:'Unknown'|variable_revision_number:2|show_window:False|notes:['To begin creating your project, use the Images module to compile a list of files and/or folders that you want to analyze. You can also specify a set of rules to include only the desired files in your selected folders.']|batch_state:array([], dtype=uint8)|enabled:True|wants_pause:False]

:

Filter images?:No filtering

Select the rule criteria:and (file does contain "slide-2_well-A3-01")

Metadata:[module_num:2|svn_version:'Unknown'|variable_revision_number:6|show_window:False|notes:['The Metadata module optionally allows you to extract information describing your images (i.e, metadata) which will be stored along with your measurements. This information can be contained in the file name and/or location, or in an external file.']|batch_state:array([], dtype=uint8)|enabled:True|wants_pause:False]

Extract metadata?:Yes

Metadata data type:Text

Metadata types:{}

Extraction method count:1

Metadata extraction method:Extract from file/folder names

Metadata source:File name

Regular expression to extract from file name:_(?P<Exp>[A-Z]{2}[0-9]{4})_63x_slide-(?P<slide>[0-9]{1})_well-(?P<well>[A-Z]{1}[0-9]{1})-(?P<Number>[0-9]{2})_0_cmle_ch(?P<channel>[0-9]{2}).tif

Regular expression to extract from folder name:(?P<Date>[0-9]{4}_[0-9]{2}_[0-9]{2})$

Extract metadata from:All images

Select the filtering criteria:and (file does contain "")

Metadata file location:Elsewhere...|

Match file and image metadata:[]

Use case insensitive matching?:No

Metadata file name:

Does cached metadata exist?:No

NamesAndTypes:[module_num:3|svn_version:'Unknown'|variable_revision_number:8|show_window:False|notes:['The NamesAndTypes module allows you to assign a meaningful name to each image by which other modules will refer to it.']|batch_state:array([], dtype=uint8)|enabled:True|wants_pause:False]

Assign a name to:Images matching rules

Select the image type:Grayscale image

Name to assign these images:image

Match metadata:[]

Image set matching method:Order

Set intensity range from:Image metadata

Assignments count:2

Single images count:0

Maximum intensity:255.0

Process as 3D?:No

Relative pixel spacing in X:1.0

Relative pixel spacing in Y:1.0

Relative pixel spacing in Z:1.0

Select the rule criteria:and (file does contain "_ch00.t")

Name to assign these images:DNA

Name to assign these objects:Cell

Select the image type:Grayscale image

Set intensity range from:Image metadata

Maximum intensity:255.0

Select the rule criteria:and (file does contain "_ch01.t")

Name to assign these images:TexRed

Name to assign these objects:Nucleus

Select the image type:Grayscale image

Set intensity range from:Image metadata

Maximum intensity:255.0

Groups:[module_num:4|svn_version:'Unknown'|variable_revision_number:2|show_window:False|notes:['The Groups module optionally allows you to split your list of images into image subsets (groups) which will be processed independently of each other. Examples of groupings include screening batches, microtiter plates, time-lapse movies, etc.']|batch_state:array([], dtype=uint8)|enabled:True|wants_pause:False]

Do you want to group your images?:No

grouping metadata count:1

Metadata category:Well

IdentifyPrimaryObjects:[module_num:5|svn_version:'Unknown'|variable_revision_number:15|show_window:False|notes:[]|batch_state:array([], dtype=uint8)|enabled:True|wants_pause:False]

Select the input image:DNA

Name the primary objects to be identified:Nuclei

Typical diameter of objects, in pixel units (Min,Max):20,400

Discard objects outside the diameter range?:Yes

Discard objects touching the border of the image?:Yes

Method to distinguish clumped objects:Intensity

Method to draw dividing lines between clumped objects:Shape

Size of smoothing filter:20

Suppress local maxima that are closer than this minimum allowed distance:20.0

Speed up by using lower-resolution image to find local maxima?:Yes

Fill holes in identified objects?:After both thresholding and declumping

Automatically calculate size of smoothing filter for declumping?:Yes

Automatically calculate minimum allowed distance between local maxima?:No

Handling of objects if excessive number of objects identified:Continue

Maximum number of objects:500

Use advanced settings?:Yes

Threshold setting version:12

Threshold strategy:Global

Thresholding method:Otsu

Threshold smoothing scale:1

Threshold correction factor:1

Lower and upper bounds on threshold:0.05,1

Manual threshold:0.34

Select the measurement to threshold with:None

Two-class or three-class thresholding?:Three classes

Log transform before thresholding?:No

Assign pixels in the middle intensity class to the foreground or the background?:Foreground

Size of adaptive window:10

Lower outlier fraction:0.05

Upper outlier fraction:0.05

Averaging method:Mean

Variance method:Standard deviation

# of deviations:2

Thresholding method:Robust Background

IdentifySecondaryObjects:[module_num:6|svn_version:'Unknown'|variable_revision_number:10|show_window:False|notes:[]|batch_state:array([], dtype=uint8)|enabled:True|wants_pause:False]

Select the input objects:Nuclei

Name the objects to be identified:Cells

Select the method to identify the secondary objects:Distance - N

Select the input image:TexRed

Number of pixels by which to expand the primary objects:270

Regularization factor:0.05

Discard secondary objects touching the border of the image?:No

Discard the associated primary objects?:No

Name the new primary objects:FilteredNuclei

Fill holes in identified objects?:Yes

Threshold setting version:12

Threshold strategy:Global

Thresholding method:Minimum Cross-Entropy

Threshold smoothing scale:1.3488

Threshold correction factor:1

Lower and upper bounds on threshold:(0.0, 1.0)

Manual threshold:0.0

Select the measurement to threshold with:None

Two-class or three-class thresholding?:Two classes

Log transform before thresholding?:No

Assign pixels in the middle intensity class to the foreground or the background?:Foreground

Size of adaptive window:10

Lower outlier fraction:0.05

Upper outlier fraction:0.05

Averaging method:Mean

Variance method:Standard deviation

# of deviations:2

Thresholding method:Default

EnhanceOrSuppressFeatures:[module_num:7|svn_version:'Unknown'|variable_revision_number:7|show_window:False|notes:[]|batch_state:array([], dtype=uint8)|enabled:True|wants_pause:False]

Select the input image:TexRed

Name the output image:FilteredTexRed

Select the operation:Enhance

Feature size:30

Feature type:Speckles

Range of hole sizes:1,10

Smoothing scale:2.0

Shear angle:0.0

Decay:0.95

Enhancement method:Tubeness

Speed and accuracy:Slow

Rescale result image:Yes

IdentifyPrimaryObjects:[module_num:8|svn_version:'Unknown'|variable_revision_number:15|show_window:False|notes:[]|batch_state:array([], dtype=uint8)|enabled:True|wants_pause:False]

Select the input image:FilteredTexRed

Name the primary objects to be identified:AllBlobs

Typical diameter of objects, in pixel units (Min,Max):3,10

Discard objects outside the diameter range?:Yes

Discard objects touching the border of the image?:Yes

Method to distinguish clumped objects:Shape

Method to draw dividing lines between clumped objects:Intensity

Size of smoothing filter:1

Suppress local maxima that are closer than this minimum allowed distance:1

Speed up by using lower-resolution image to find local maxima?:Yes

Fill holes in identified objects?:Never

Automatically calculate size of smoothing filter for declumping?:No

Automatically calculate minimum allowed distance between local maxima?:No

Handling of objects if excessive number of objects identified:Continue

Maximum number of objects:500

Use advanced settings?:Yes

Threshold setting version:12

Threshold strategy:Adaptive

Thresholding method:Robust Background

Threshold smoothing scale:1.3488

Threshold correction factor:1.0

Lower and upper bounds on threshold:0.4,1.0

Manual threshold:0.08

Select the measurement to threshold with:None

Two-class or three-class thresholding?:Three classes

Log transform before thresholding?:No

Assign pixels in the middle intensity class to the foreground or the background?:Foreground

Size of adaptive window:10

Lower outlier fraction:0.06

Upper outlier fraction:0.06

Averaging method:Mean

Variance method:Standard deviation

# of deviations:2

Thresholding method:Robust Background

MaskObjects:[module_num:9|svn_version:'Unknown'|variable_revision_number:3|show_window:False|notes:[]|batch_state:array([], dtype=uint8)|enabled:True|wants_pause:True]

Select objects to be masked:AllBlobs

Name the masked objects:BlobsInCells

Mask using a region defined by other objects or by binary image?:Objects

Select the masking object:Cells

Select the masking image:BinaryCy5

Handling of objects that are partially masked:Keep

Fraction of object that must overlap:0.5

Numbering of resulting objects:Renumber

Invert the mask?:No

RelateObjects:[module_num:10|svn_version:'Unknown'|variable_revision_number:5|show_window:False|notes:[]|batch_state:array([], dtype=uint8)|enabled:True|wants_pause:False]

Parent objects:Cells

Child objects:BlobsInCells

Calculate child-parent distances?:None

Calculate per-parent means for all child measurements?:Yes

Calculate distances to other parents?:No

Do you want to save the children with parents as a new object set?:No

Name the output object:RelateObjects_Atto647

Parent name:None

Parent name:None

Parent name:None

Parent name:None

Parent name:None

Parent name:None

Parent name:None

Parent name:None

Parent name:None

Parent name:None

Parent name:None

Parent name:None

Parent name:None

Parent name:None

Parent name:None

Parent name:None

Parent name:None

Parent name:None

Parent name:None

Parent name:None

Parent name:None

Parent name:None

Parent name:None

Parent name:None

ExportToSpreadsheet:[module_num:11|svn_version:'Unknown'|variable_revision_number:13|show_window:False|notes:[]|batch_state:array([], dtype=uint8)|enabled:True|wants_pause:True]

Select the column delimiter:Comma (",")

Add image metadata columns to your object data file?:No

Add image file and folder names to your object data file?:No

Select the measurements to export:Yes

Calculate the per-image mean values for object measurements?:No

Calculate the per-image median values for object measurements?:No

Calculate the per-image standard deviation values for object measurements?:No

Output file location:Default Output

Create a GenePattern GCT file?:No

Select source of sample row name:Metadata

Select the image to use as the identifier:None

Select the metadata to use as the identifier:None

Export all measurement types?:Yes

Press button to select measurements:Image|ExecutionTime_10MaskObjects,Image|ExecutionTime_02Metadata,Image|ExecutionTime_11RelateObjects,Image|ExecutionTime_06IdentifySecondaryObjects,Image|ExecutionTime_04Groups,Image|ExecutionTime_01Images,Image|ExecutionTime_07

EnhanceOrSuppressFeatures,Image|ExecutionTime_05IdentifyPrimaryObjects,Image|ExecutionTime_09IdentifyPrimaryObjects,Image|ExecutionTime_03NamesAndTypes,Image|PathName_DNA,Image|PathName_TexRed,Image|Width_TexRed,Image|Width_DNA,Image|MD5Digest_TexRed,Image|MD5Digest_DNA,Image|Threshold_OrigThreshold_AllBlobs,Image|Threshold_OrigThreshold_Nuclei,Image|Threshold_WeightedVariance_AllBlobs,Image|Threshold_WeightedVariance_Nuclei,Image|Threshold_FinalThreshold_Nuclei,Image|Threshold_FinalThreshold_AllBlobs,Image|Threshold_SumOfEntropies_AllBlobs,Image|Threshold_SumOfEntropies_Nuclei,Image|Threshold_GuideThreshold_AllBlobs,Image|Metadata_Frame,Image|Metadata_well,Image|Metadata_channel,Image|Metadata_slide,Image|Metadata_Series,Image|Metadata_Number,Image|Metadata_Exp,Image|Metadata_FileLocation,Image|Height_DNA,Image|Height_TexRed,Image|Scaling_DNA,Image|Scaling_TexRed,Image|Count_Nuclei,Image|Count_BlobsInCells,Image|Count_AllBlobs,Image|Count_Cells,Image|FileName_DNA,Image|FileName_TexRed,Image|ModuleError_01Images,Image|ModuleError_11RelateObjects,Image|ModuleError_04Groups,Image|ModuleError_09IdentifyPrimaryObjects,Image|ModuleError_07EnhanceOrSuppressFeatures,Image|ModuleError_06IdentifySecondaryObjects,Image|ModuleError_10MaskObjects,Image|ModuleError_05IdentifyPrimaryObjects,Image|ModuleError_03NamesAndTypes,Image|ModuleError_02Metadata,Image|Frame_TexRed,Image|Frame_DNA,Image|Group_Number,Image|Group_Index,Image|Series_TexRed,Image|Series_DNA,Image|URL_TexRed,Image|URL_DNA,Cells|Location_Center_Z,Cells|Location_Center_X,Cells|Location_Center_Y,Cells|Mean_BlobsInCells_Location_Center_Y,Cells|Mean_BlobsInCells_Location_Center_X,Cells|Mean_BlobsInCells_Number_Object_Number,Cells|Number_Object_Number,Cells|Children_BlobsInCells_Count,Cells|Parent_Nuclei,AllBlobs|Children_BlobsInCells_Count,AllBlobs|Location_Center_Y,AllBlobs|Location_Center_X,AllBlobs|Location_Center_Z,AllBlobs|Number_Object_Number,Experiment|Run_Timestamp,Experiment|Modification_Timestamp,Experiment|CellProfiler_Version,Experiment|Pipeline_Pipeline,BlobsInCells|Location_Center_Y,BlobsInCells|Location_Center_X,BlobsInCells|Number_Object_Number,BlobsInCells|Parent_Cells,BlobsInCells|Parent_AllBlobs,Nuclei|Children_Cells_Count,Nuclei|Number_Object_Number,Nuclei|Location_Center_Y,Nuclei|Location_Center_Z,Nuclei|Location_Center_X

Representation of Nan/Inf:NaN

Add a prefix to file names?:Yes

Filename prefix:

Overwrite existing files without warning?:No

Data to export:Experiment

Combine these object measurements with those of the previous object?:No

File name:DATA.csv

Use the object name for the file name?:Yes

Data to export:Image

Combine these object measurements with those of the previous object?:No

File name:DATA.csv

Use the object name for the file name?:Yes

Data to export:Cells

Combine these object measurements with those of the previous object?:No

File name:DATA.csv

Use the object name for the file name?:Yes

Data to export:BlobsInCells

Combine these object measurements with those of the previous object?:No

File name:DATA.csv

Use the object name for the file name?:Yes

Supplementary References

1. Joffroy, B., Uca, Y. O., Presern, D., Doye, J. P. K. & Schmidt, T. L. Rolling Circle Amplification shows a sinusoidal length-dependent amplification bias. *Nucleic Acids Res.* **46**, 538–545 (2018).
2. Mick, D. U., Fox, T. D. & Rehling, P. Inventory control: cytochrome c oxidase assembly regulates mitochondrial translation. *Nat. Rev. Mol. Cell Biol.* **12**, 14–20 (2011).
3. García-Villegas, R. *et al.* The Cox1 C-terminal domain is a central regulator of cytochrome c oxidase biogenesis in yeast mitochondria. *J. Biol. Chem.* **292**, 10912–10925 (2017).
4. Nakamura, N. *et al.* Characterization of a cis-Golgi matrix protein, GM130. *J. Cell Biol.* **131**, 1715–1726 (1995).
5. Uhlén, M. *et al.* Tissue-based map of the human proteome. *Science (80-.).* **347**, (2015).
6. Gubbels, J. A. *et al.* Mesothelin-MUC16 binding is a high affinity, N-glycan dependent interaction that facilitates peritoneal metastasis of ovarian tumors. *Mol. Cancer* **5**, 1476–4598 (2006).
7. Coelho, R. *et al.* Peritoneal dissemination of ovarian cancer: role of MUC16-mesothelin interaction and implications for treatment. *Expert Rev. Anticancer Ther.* **18**, (2018).

Original Images for Blots, PDIA3 knock-down (Fig. 7, Suppl. Fig. 5)

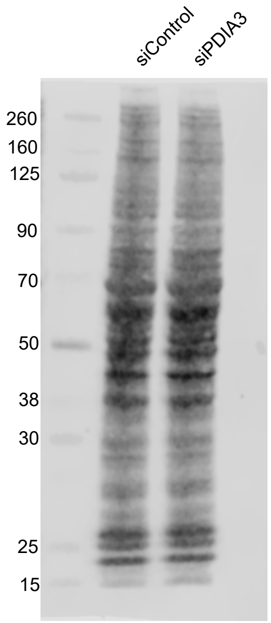

Fig. 7, blot 1, channel 1
Total protein

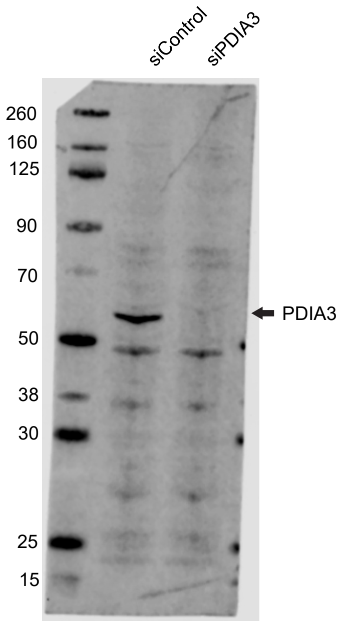

Fig. 7, blot 1,channel 2
